# Discovery of lncRNA‐Based ProsRISK Score in Serum as Potential Biomarkers for Improved Accuracy of Prostate Cancer Detection

**DOI:** 10.1111/jcmm.70555

**Published:** 2025-04-21

**Authors:** Xiumei Jiang, Zhongchao Liu, Hongxing Wang, Lishui Wang

**Affiliations:** ^1^ Department of Clinical Laboratory, Qilu Hospital Shandong University Jinan Shandong Province People's Republic of China

**Keywords:** biomarker, diagnosis, lncRNA, prostate cancer, serum

## Abstract

Circulating lncRNAs have emerged as promising biomarkers for the diagnosis of various cancers. This study aimed to establish an accurate risk prediction model based on serum lncRNAs to facilitate the detection of prostate cancer (PCA). RT‐qPCR was used to analyse the levels of candidate lncRNAs, and four lncRNAs (NEAT1, ARLNC1, FOXP4‐AS1 and DSCAM‐AS1) were identified to be differently expressed in serum from 190 PCA patients, 140 benign controls, and 170 healthy controls. A ProsRISK score based on four lncRNAs and prostate‐specific antigen (PSA) was established in the training set. ROC analysis in the validation set revealed that the ProsRISK demonstrated more powerful capacity in discriminating PCA from healthy and benign controls, with an AUC of 0.926 (95% CI: 0.882–0.970) and 0.837 (95% CI: 0.770–0.904), which were significantly higher than those of the lncRNA panel or PSA alone (all at *p* < 0.05). Moreover, the ProsRISK showed good diagnostic performance for PCA I–II patients compared with healthy and benign controls, and the corresponding AUCs were 0.905 (95% CI: 0.843–0.968) and 0.819 (95% CI: 0.732–0.907). Our findings indicated that the constructed ProsRISK could be a reliable risk stratification model and have great potential for clinical use to improve the precision surveillance for PCA.

## 1 Introduction

Prostate cancer (PCA) is the most common malignancy of the genitourinary system and the second leading cause of male cancer‐related death worldwide [1]. It is estimated that the global burden of PCA will grow to almost 2.3 million new cases and 740,000 deaths by 2040 [2]. In China, the incidence and mortality rate of PCA have also been increasing in recent years [3]. As patients in early stages generally have no typical symptoms, approximately 10% of patients showed evidence of locally advanced cancer and 5% present with distant metastasis at initial diagnosis [4]. Currently, serum prostate‐specific antigen (PSA) and digital rectal exam (DRE) are commonly used screening tools for PCA. Despite PSA having a relatively good sensitivity (SN) as it is secreted by prostate cells, its specificity (SP) is low due to the fact that many benign conditions can also cause elevated PSA levels [5, 6]. In the case of DRE, it could only assess peripheral zone tumours and its diagnostic performance is also poor [7]. Although prostate biopsy is the gold standard tool for diagnosis, it is invasive and the possibility of false negatives makes patients undergo repeated biopsies under MRI guidance [8]. These limitations highlight the urgent need for minimally invasive biomarkers with improved SN and SP for PCA.

Long noncoding RNAs (lncRNAs) are a group of endogenous RNA molecules with a length of longer than 200 nucleotides that regulate gene expression at the posttranscriptional level [9]. Numerous lncRNAs are reported to be dysregulated and play key roles in the initiation and progression of cancer, including PCA [10]. Accumulating evidence showed that profiling changes of lncRNAs acting as oncogenes or tumour suppressor genes could exist in the serum of cancer [11, 12]. In PCA, research has been undertaken to explore the serum expression levels of lncRNAs that are involved in the pathogenesis of cancer. For instance, Hu et al. [13] found that MAGI2‐AS3 could inhibit cancer progression by targeting miR‐142‐3p and was down‐regulated in the serum of PCA. Moreover, Zhao et al. [14] revealed that serum MALAT1 and TMPRSS2‐ETV1 could aid in evaluating the progress and prognosis of PCA. However, knowledge is still limited regarding the expression of circulating lncRNAs in PCA patients and their potential utilities as routine diagnostic biomarkers for clinical use.

In the present study, we explored the expression profile of lncRNAs by RT‐qPCR assays in serum samples of PCA patients, healthy controls, and symptomatic patients with benign prostate diseases, which represent a challenge in the early detection of PCA because of the overlapping symptoms. A ProsRISK score based on the differentially expressed lncRNAs and PSA was constructed and validated using two independent cohorts. The obtained SN and SP data were used to identify the most appropriate cut‐offs for the ProsRISK, which enables stratification of patients into those with normal or elevated risk for developing PCA.

## 2 Materials and Methods

### 2.1 Ethical Statement

This study was approved by the Clinical Research Ethics Committee of Qilu Hospital, Shandong University. All procedures included in the study involving human participants were carried out according to the ethical standards of the Clinical Research Ethics Committee of Qilu Hospital and the Helsinki Declaration. Clinical samples were collected from the Qilu Hospital of Shandong University and written informed consent was obtained from every participant.

### 2.2 Patients and Control Subjects

In all, 190 patients with PCA and 140 patients with benign prostate disease, including benign prostate hyperplasia (BPH) and prostatitis, were enrolled in this study, and blood samples were collected one day before prostate biopsy in the Department of Urology of Qilu Hospital, Shandong University. Totally 20 pairs of PCA tissues and adjacent tissues were collected from patients who underwent surgical resection. Meanwhile, 60 patients with other common urological tract malignancies, consisting of 30 patients with renal cell carcinoma (RCC) and 30 patients with bladder transitional cell cancer (TCC) were also recruited. All patients were not treated with preoperative therapy and were excluded from other tumour diseases. Histological specimens from all patients were examined to ensure the diagnosis of cancer or benign diseases. In addition, 170 healthy participants seeking medical check‐up in the Healthy Physical Examination Center of Qilu Hospital, Shandong University, who showed no evidence of disease, were selected as healthy controls. The clinical features of participants in this study, including age, preoperative PSA level, Gleason score, tumour stage, lymph node metastasis and bone metastasis, were retrospectively collected (Table S1).

### 2.3 Study Design

The present study was divided into three phases (Figure S1). In the discovery phase, considering that down‐regulated lncRNAs are not easily detectable because of their low expression level, we focused on up‐regulated lncRNAs in PCA revealed by validation of the top 20 lncRNAs in PCA in the TCGA database and 20 most relevant lncRNAs with PCA in PubMed using 20 paired PCA/adjacent normal tissues [15, 16]. In the training phase, candidate lncRNAs were initially measured in serum from 30 PCA patients, 30 benign patients and 30 healthy controls. Four differentially expressed lncRNAs among these three groups were selected. We expanded the sample size to 120 PCA patients, 80 benign patients, and 110 healthy controls and confirmed the differential expression patterns. A Logistic regression analysis was then applied and a ProsRISK score was created using the 5 predictors: 4 lncRNAs and PSA. In the validation phase, we evaluated the diagnostic values of the ProsRISK in another independent cohort including 70 PCA patients, 60 benign patients and 60 healthy controls. In order to select the most appropriate cut‐offs for the ProsRISK score, we explored the dynamic changes in SN and SP after presetting their values and constructed 2 versions of ProsRISK (ProsRISK‐Fam and ProsRISK‐Sym). Finally, the stability and specificity of these lncRNAs were analysed by measuring their expression under harsh conditions and in other urological tract malignancies.

### 2.4 Sample Collection and Preparation

Peripheral blood samples (5 mL) were collected from all subjects for serum extraction. Sequential centrifugation (4000 rpm for 10 min at 4°C, 12,000 rpm for 15 min) within 2 h of blood collection was carried out to completely remove cell debris. The serum samples were stored in RNase/DNase‐free tubes at −80°C until use. Fresh tumour tissues and adjacent noncancerous tissues were immediately frozen in liquid nitrogen and stored at −80°C.

### 2.5 Quantification of lncRNAs by RT‐qPCR Assays

Briefly, total RNA was isolated from serum and tissues using TRIzol LS and TRIzol (Invitrogen, Carlsbad, CA, USA). RT reactions were performed using the PrimeScript RT reagent kit (Takara, Dalian, China). Then, cDNA generated from RT reactions was submitted to quantitative PCR analysis using a CFX96 Real‐Time PCR Detection System (Bio‐Rad Laboratories, Hercules, CA, USA). The reaction reagents contained 2 μL of cDNA, 12.5 μL of SYBR Premix Ex Taq, 0.5 μL of ROX Reference Dyeα, 1 μL of forward primer, 1 μL of reverse primer, and 8 μL of RNase‐free dH_2_O. The reaction parameters were continuous incubation at 95°C for 30s, followed by 42 cycles of 95°C for 5 s and 60°C for 34 s. GAPDH was used as the endogenous control. Primers used for RT‐qPCR assays of lncRNAs are presented in Table S2. The mathematical model of 2^−∆∆Ct^ method was applied to calculate the expression level of lncRNAs.

### 2.6 Statistical Analysis

Kolmogorov–Smirnov test was employed to measure the data distribution of each group. Nonparametric Mann–Whitney U test was conducted for comparison of lncRNA expression between different groups. Logistic regression analysis was performed utilising MATLAB software (MATLAB R2014a). The performance characteristics of the ProsRISK were assessed and compared in terms of area under the ROC curve (AUC) using MedCalc 9.3.9.0. The SP at fixed SN and SN at fixed SP were respectively used in the analysis of control versus PCA as well as benign versus PCA. Performance of the ProsRISK was separately evaluated at clinically relevant SN cut‐offs and SP cutoffs for the detection of asymptomatic and symptomatic individuals (both set at > 80%) [17]. Positive and negative predictive values (PPV and NPV) for a number of prevalence estimates were calculated according to the standard approach [18]. SPSS software (version 18.0, Chicago, IL, USA) and GraphPad Prism (version 8) were used to analyse all other data and graph plots. The *p* < 0.05 was considered statistically significant.

## 3 Results

### 3.1 Selection and Evaluation of Candidate lncRNAs in Serum of PCA

**FIGURE 1:**
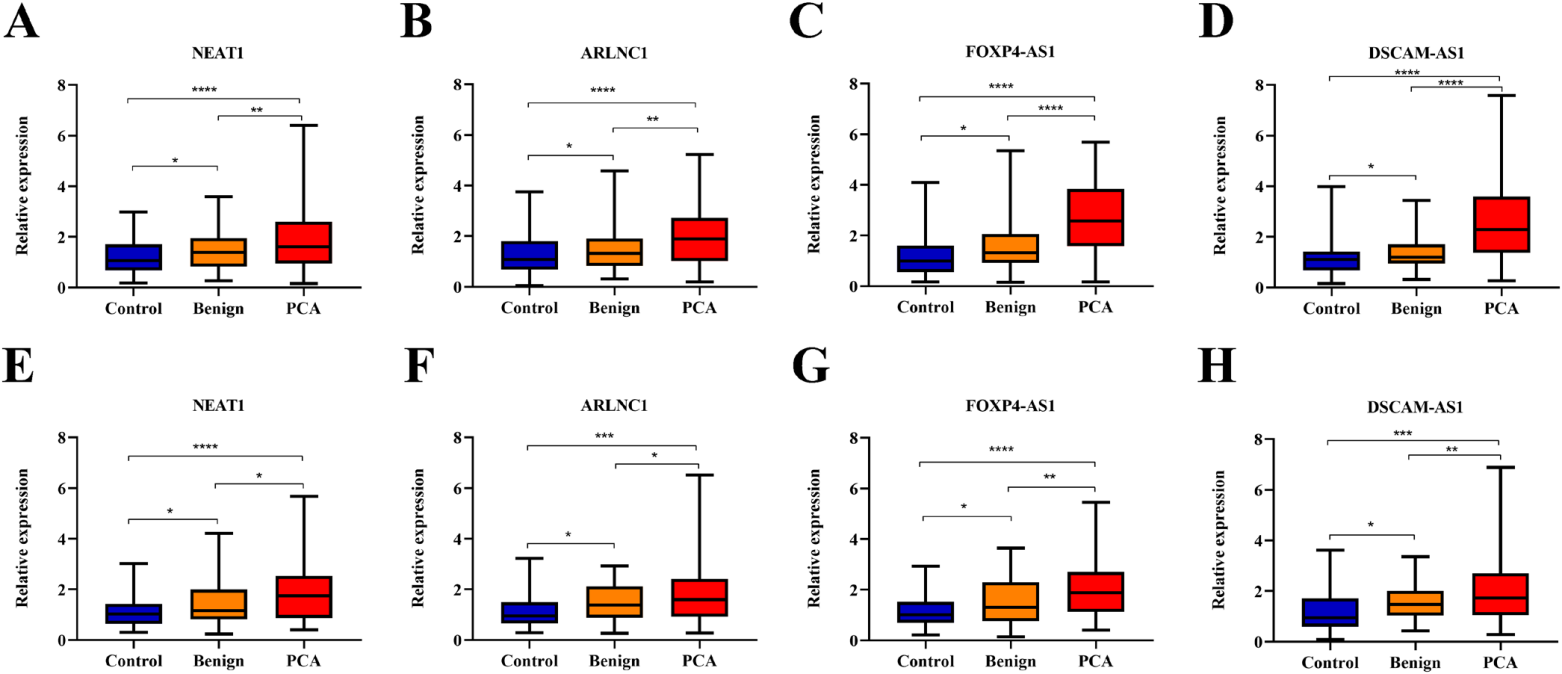
Box‐whisker plots represent the expression of four selected lncRNAs in the serum of patients with PCA, benign controls and healthy controls using RT‐qPCR in the training set (A–D) and validation set (E–H), ****p < 0.0001, ***p < 0.001, **p < 0.01, *p < 0.05.

Initially, RNA sequencing data of 501 PCA tissues and 52 healthy tissues were downloaded from the TCGA database and the top 20 lncRNAs with the fold change ≥ 2 and *p* < 0.05 were selected (Table S3). Meanwhile, the 20 most potential lncRNAs with vital biological values for PCA from literature review in PubMed were obtained (Table S4). Taken together, 35 lncRNAs were revealed and subjected to RT‐qPCR assays using 20 paired PCA/adjacent normal tissues (Table S5). We found that 14 lncRNAs were significantly up‐regulated in PCA (Figure S2). In accordance with our findings, 10 of 14 lncRNAs were significantly up‐regulated in PCA in the TCGA database (Figure S3), whereas 3 lncRNAs showed similar expression patterns and 1 lncRNA was down‐regulated, which may be influenced by heterogeneity, irregularity and other characteristics [19]. Thus, these 14 lncRNAs were selected to be further detected in serum of 30 PCA patients, 30 benign controls and 30 healthy controls by RT‐qPCR. With the filter criteria of *p* < 0.05 and lncRNAs with Ct value of < 35 and detection rate of > 75%, four lncRNAs (NEAT1, ARLNC1, FOXP4‐AS1 and DSCAM‐AS1) were found to be differentially expressed among the three groups (Figure S4). We then expanded the sample size to 120 patients with PCA, 80 benign controls and 110 healthy controls and confirmed the above phenomenon in the training set (Table 1, Figure 1A–D). In differentiating PCA from healthy controls, ROC analysis revealed that the corresponding AUCs of four lncRNAs were 0.684 (95% confidence interval [CI]: 0.616–0.752), 0.689 (95% CI: 0.622–0.757), 0.807 (95% CI: 0.750–0.863) and 0.809 (95% CI: 0.753–0.865), respectively (Figure S5A–D). In differentiating PCA from benign controls, the corresponding AUCs of the four lncRNAs were 0.596 (95% CI: 0.516–0.675), 0.616 (95% CI: 0.538–0.694), 0.736 (95% CI: 0.667–0.806) and 0.751 (95% CI: 0.685–0.818) respectively (Figure S5E–H).

**TABLE 1:** Relative expressions of 4 candidate lncRNAs in serum of patients with PCA and controls in the training set and validation set.

LncRNA	Training set			Validation set		
	Healthy controls (*n* = 110)	Benign controls (*n* = 80)	PCA patients (*n* = 120)	Healthy controls (*n* = 60)	Benign controls (*n* = 60)	PCA patients (*n* = 70)
NEAT1	1.06 (0.68–1.71)	1.39 (0.83–1.95)	1.61 (0.95–2.60)	1.02 (0.64–1.42)	1.15 (0.82–2.00)	1.75 (0.87–2.54)
ARLNC1	1.07 (0.68–1.81)	1.32 (0.82–1.91)	1.89 (1.01–2.72)	0.96 (0.66–1.50)	1.37 (0.88–2.11)	1.60 (0.93–2.41)
FOXP4‐AS1	1.00 (0.56–1.60)	1.32 (0.92–2.06)	2.57 (1.58–3.84)	1.01 (0.70–1.52)	1.31 (0.76–2.29)	1.88 (1.13–2.69)
DSCAM‐AS1	1.10 (0.68–1.43)	1.20 (0.95–1.71)	2.28 (1.37–3.59)	0.94 (0.59–1.71)	1.47 (1.03–2.00)	1.72 (1.05–2.70)

*Note:* Data are presented as median (interquartile range).

To further verify the accuracy of these four lncRNAs, we assessed their expression levels in another independent cohort including 70 patients with PCA, 60 benign controls and 60 healthy controls, and the expression profiles of these four lncRNAs in the validation set were similar to those in the training set (Figure 1E–H), with AUCs respectively ranging from 0.675 to 0.735 and 0.583 to 0.640 for discriminating PCA from healthy and benign controls (Figure S6).

### 3.2 Diagnostic Values of the lncRNA‐Based Panel in Serum for PCA

**FIGURE 2:**
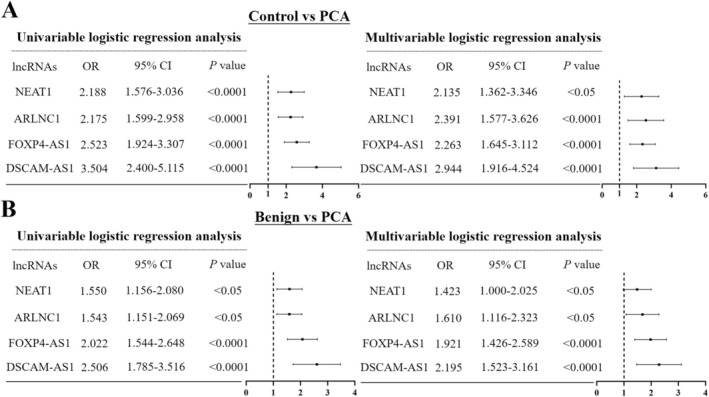
Forest plot summary of the univariate and multivariate logistic regression analysis of four lncRNAs for diagnosis of PCA from healthy controls (A) and benign controls (B) in the training set. The squares on the transverse lines represent the odds ratio (OR), and the transverse lines represent the 95% CI.

**FIGURE 3:**
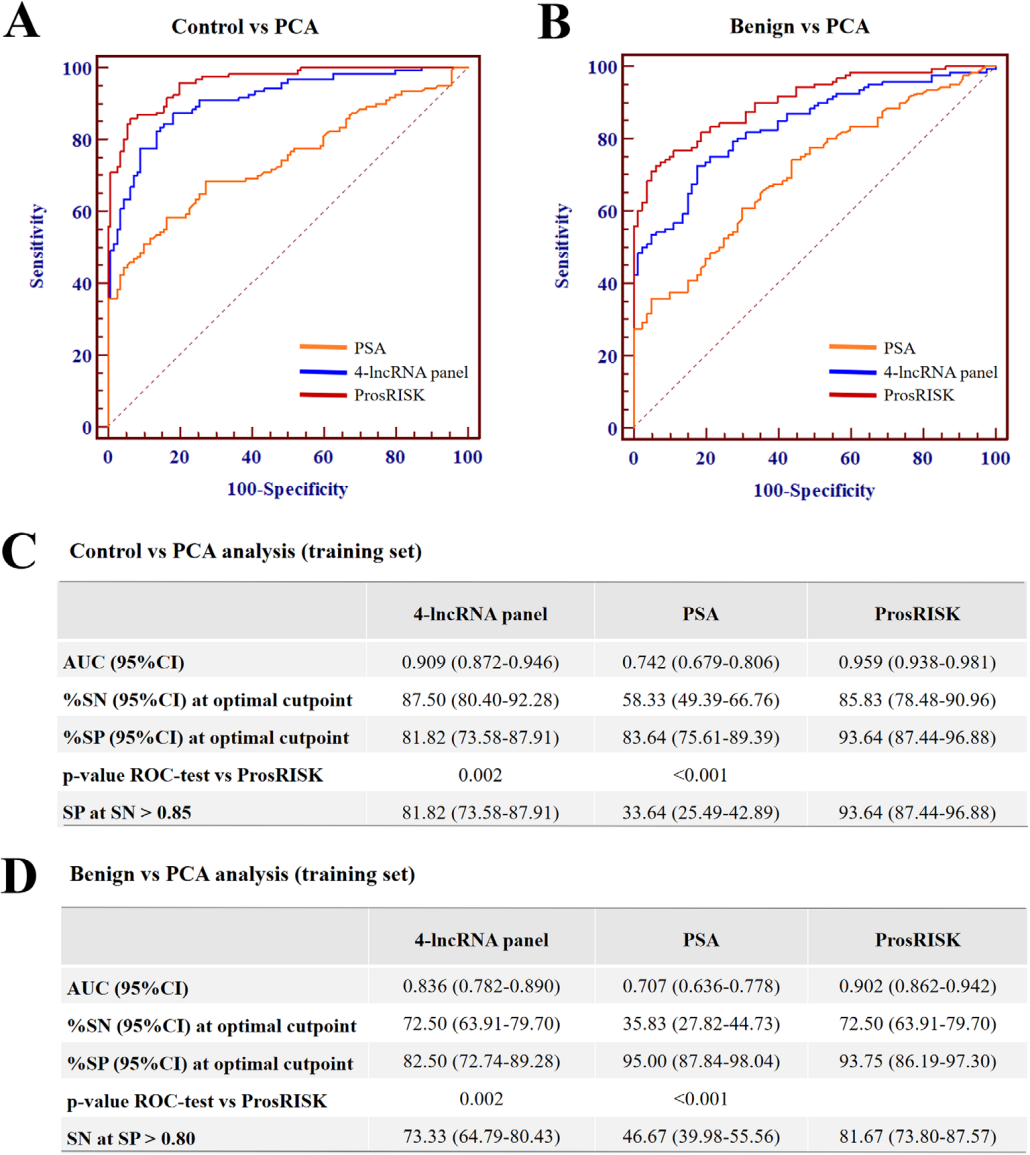
Evaluation of the ProsRISK for PCA discrimination in the training set. Performance in distinguishing PCA from healthy controls (A and C) and benign controls (B and D). AUC, area under the receiver operating characteristic curve; CI, confidence interval; SN, sensitivity; SP, specificity.

By logistic regression analysis, four lncRNAs were identified as independent factors for the diagnosis of PCA (Figure 2) and a 4‐lncRNA panel was constructed using the training cohort. For detecting PCA from healthy and benign controls, the 4‐lncRNA panel yielded an AUC of 0.909 (95% CI: 0.872–0.946) and 0.836 (95% CI: 0.782–0.890), respectively (Figure 3). Meanwhile, the diagnostic performance of PSA was also assessed and the corresponding AUCs for differentiating PCA from healthy and benign controls were 0.742 (95% CI: 0.679–0.806) and 0.707 (95% CI: 0.636–0.778) (Figure 3).

### 3.3 Establishment of the ProsRISK for Diagnosis of PCA

The model that incorporated four lncRNA and PSA was developed and presented as a ProsRISK score in the training set. The performance characteristics by ROC curve analysis revealed that the ProsRISK resulted in an AUC of 0.959 (95% CI: 0.938–0.981) and 0.902 (95% CI: 0.862–0.942) for the diagnosis of PCA in comparisons with healthy samples and benign samples, significantly higher than those of the 4‐lncRNA panel or PSA alone (all at *p* < 0.05, Figure 3).

### 3.4 Validation of the ProsRISK for Differentiating Control Individuals and Those With Benign Conditions From PCA Patients

**FIGURE 4:**
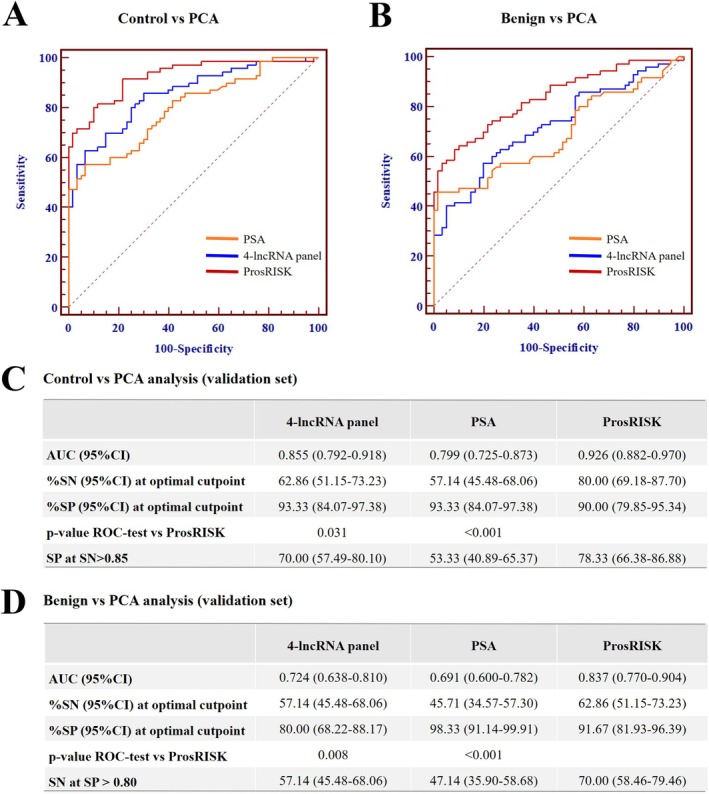
Evaluation of the ProsRISK for PCA discrimination in the validation set. Performance in distinguishing PCA from healthy controls (A and C) and benign controls (B and D).

**FIGURE 5:**
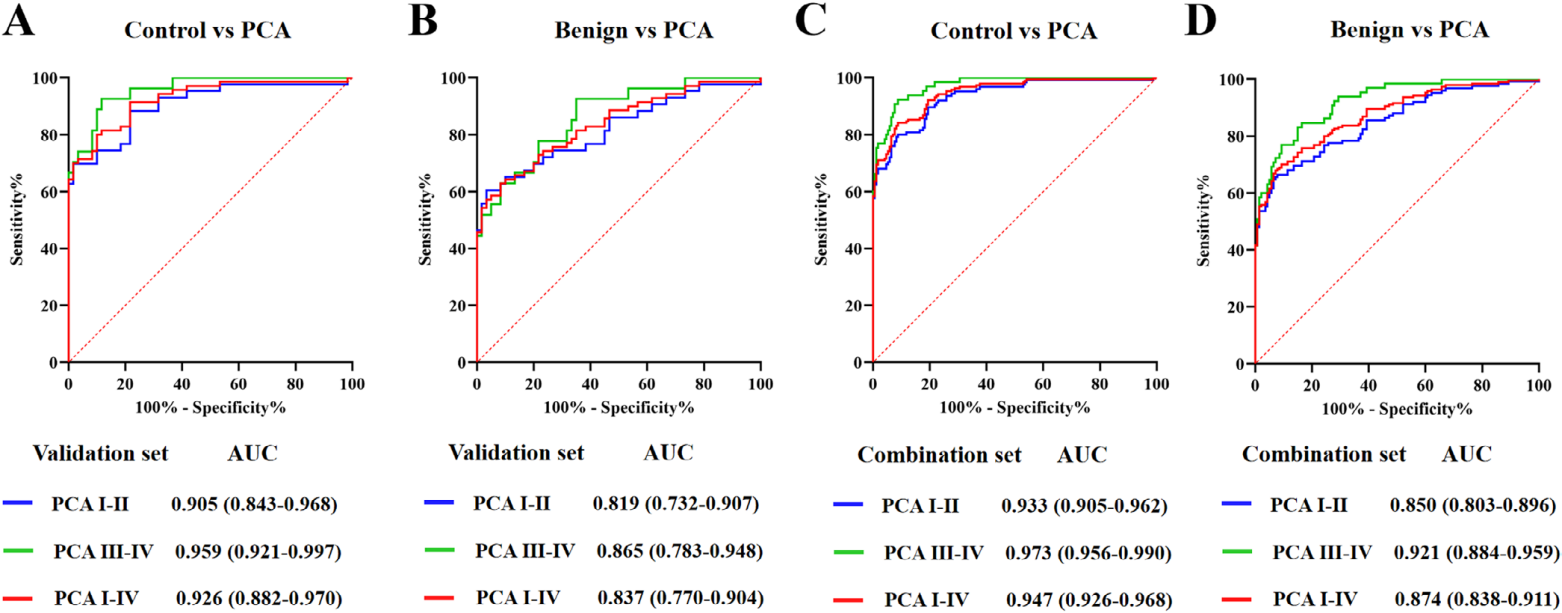
ROC curve analysis for comparison of the ProsRISK for different stages of PCA with healthy controls (A and C) and benign controls (B and D) in the validation set and combination set.

In agreement with the training set, favourable calibration of the ProsRISK was confirmed in the validation set with an AUC of 0.926 (95% CI: 0.882–0.970) and 0.837 (95% CI: 0.770–0.904) for PCA when compared with healthy and benign controls (Figure 4). Similarly, the ProsRISK outperformed the 4‐lncRNA panel and PSA with regard to detecting PCA (all at *p* < 0.05). For discriminating PCA stages I–II from healthy and benign controls, the corresponding AUCs were 0.905 (95% CI: 0.843–0.968) and 0.819 (95% CI: 0.732–0.907), which indicated well performance of the ProsRISK in monitoring early stage tumours (Figure 5A,B). We further pooled participants from the training set and validation set, and the combined results showed that the ProsRISK could detect PCA from healthy and benign groups with an AUC of 0.947 (95% CI: 0.926–0.968) and 0.874 (95% CI: 0.838–0.911) (Figure 5C,D).

With the aim to identify the most appropriate cut‐offs for the ProsRISK score, we analysed the corresponding SP at fixed SN in comparison between healthy controls and PCA as well as SN at fixed SP in comparison of benign controls with PCA (Table S6 and Table S7). Thus, two versions of ProsRISK (ProsRISK‐Fam and ProsRISK‐Sym) were established. ProsRISK‐Fam was constructed to stratify the asymptomatic patients at risk (familial history, genetic syndromes), for which we therefore set the SN cut‐off at 0.85 so as to detect the malignant cases. ProsRISK‐Sym was used for stratification of symptomatic patients and we have preset the SP cut‐off at 0.80. The patients with ‘normal’ risk should not be further investigated whereas the ones with ‘elevated’ risk should undergo more detailed examinations.

Due to the accurate prevalence of PCA still being unclear, PPV and NPV for a number of prevalence estimates were analysed in the validation set for PCA compared with healthy controls (Table S8) and for PCA compared with benign controls (Table S9). A high NPV, excluding the majority of patients with benign diseases that would show a ‘normal’ risk for PCA, was exhibited.

### 3.5 Subset Analysis of Performance of the ProsRISK in Differentiating PCA From BPH

The performance of the ProsRISK in discriminating patients with PCA from 91 patients with BPH, a subset of the benign prostate disease samples, was calculated using the training and validation datasets. The ProsRISK resulted in an AUC of 0.897 (95% CI 0.851–0.942) and 0.837 (95% CI 0.765–0.910) in the training and validation sets (Figure S7). Comparisons by ROC curve analysis also revealed better performance of ProsRISK than lncRNA‐based panel and PSA alone in discriminating PCA from BPH (Figure S8).

### 3.6 Confirmation of Identified lncRNAs Stability in Serum

In order to explore a robust biomarker panel for future clinical use, we investigated the stability of NEAT1, ARLNC1, FOXP4‐AS1 and DSCAM‐AS1 in serum samples from five donors. These serum samples were subjected to room temperature incubation (4, 8 and 24 h) and harsh conditions, including incubation at −80°C for 1, 2 and 3 months, or 1, 2 and 4 repetitive freeze–thaw cycles. RT‐qPCR analysis demonstrated that when incubated at room temperature for 4, 8 and 24 h, the expression levels of serum four lncRNAs remained stable. Moreover, prolonged incubation at −80°C or repetitive freeze–thaw cycles also had no effect on expression levels of four lncRNAs (Figure S9A–D). Our data indicated that lncRNAs were sufficiently stable in serum and they could be used for routine processing of clinical samples for tumour diagnosis.

### 3.7 Evaluation of Serum lncRNA Expression in Other Urinary Tract Cancers

**FIGURE 6:**
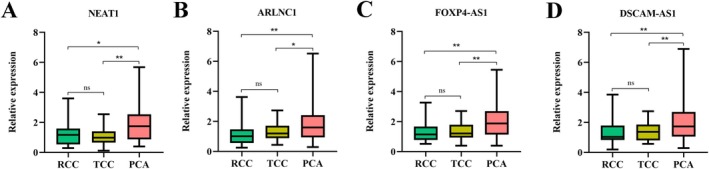
Expression levels of four lncRNAs in common genitourinary cancers by RT‐qPCR assays. **p < 0.01, *p < 0.05; ns, not significant.

Finally, we tested the expression of four lncRNAs in the serum of other common urinary tract cancers, including 30 RCC and 30 TCC. We found that all four of these lncRNAs were statistically up‐regulated in PCA compared with RCC and TCC, but no significant differences were found in expression levels between RCC and TCC (Figure 6). These data illustrated the overall good SP of these four lncRNAs as potential biomarkers for PCA detection.

## 4 Discussion

In this study, we for the first time reported on the construction of the ProsRISK score comprising four up‐regulated lncRNAs (NEAT1, ARLNC1, FOXP4‐AS1 and DSCAM‐AS1) and PSA in serum, and demonstrated its robust diagnostic accuracy in discriminating patients with PCA from healthy individuals and patients with benign prostate diseases. We also showed that these lncRNAs are stable in serum and have well SP for the detection of PCA compared with other common urinary tract cancers.

LncRNAs have recently drawn considerable attention because of their vast and pervasive impacts on cancer. All four lncRNAs revealed in this study have been reported to be up‐regulated in solid tumours and involved in the pathogenesis of PCA. NEAT1, known as nuclear‐enriched abundant transcript 1, functions as a mediation factor for transcription [20] and is overexpressed in several types of cancer [21, 22]. In PCA, it also plays vital roles in promoting development [23] and bone metastasis [24]. ARLNC1 (androgen receptor‐regulated long noncoding RNA 1) has been shown to exhibit a highly specific expression pattern in tissues of PCA and is strongly associated with AR signalling in cancer progression [25]. Additionally, FOXP4‐AS1 (forkhead box P4 antisense RNA 1) participates in multiple pathological activities of cancer [26, 27] and exerts its oncogenic functions via regulating FOXP4 and miR‐3184‐5p in PCA [28]. Furthermore, DSCAM‐AS1 (DSCAM Antisense RNA 1) has been found to play carcinogenic roles in different types of cancer [29] and could promote the progression of PCA by interacting with YBX1 [30]. These researches on the molecular functions of four lncRNAs may contribute to investigating the complicated mechanisms of tumourigenesis and provide new thoughts in exploring novel diagnostic biomarkers for PCA.

Among diverse cancer‐derived circulating materials, extracellular lncRNAs have been shown to have great potential serving as compelling components to investigate cancer‐related alterations [31]. It is speculated that circulating lncRNAs might be derived from passive secretion from necrosis/apoptosis cells or active secretion from cancer cells via membrane‐bound vesicles or binding with protein complexes, suggesting lncRNAs as suitable biomarker candidates for diagnostic test [32, 33]. Previous studies by us have shown that lncRNA‐based panels in serum have considerable value for the diagnosis of cancer [34, 35]. Focusing on lncRNAs with up‐regulated expression and functional roles in tumour tissues/cells could contribute to the evaluation of serum lncRNAs as biomarkers for cancer [34, 36]. In this study, we selected the highly expressed lncRNAs in tissues of PCA and examined their expression in serum of PCA patients as well as healthy individuals and patients with benign prostate diseases. Four lncRNAs were finally identified as differentially expressed. A ProsRISK score based on four lncRNAs and PSA was constructed and demonstrated higher diagnostic accuracy for PCA compared with the lncRNA panel or PSA alone. We also showed that these lncRNAs were robustly and stably expressed in serum, which was in line with previous studies [37]. These findings were crucial for the development of noninvasive assays based on serum lncRNAs for PCA detection with the aim of future translation into the clinical setting.

Among the identified lncRNAs, serum NEAT1 has been shown to be promising diagnostic and prognostic biomarkers for other types of cancer including colorectal cancer [38], hepatocellular carcinoma [39] and breast cancer [40]. In this study, we firstly verified that NEAT1, ARLNC1, FOXP4‐AS1, and DSCAM‐AS1 were significantly highly expressed in the serum of PCA. Despite that serum MALAT1 and TMPRSS2‐ETV1 were reported to be indicators for progression and prognosis of PCA [14], TMPRSS2‐ETV1 was not selected for validation because it has also been found to be not detectable in PCA [41]. RT‐qPCR analysis in our study revealed no significant differences in the expression of MALAT1 between PCA and healthy or benign controls. These inconsistencies may be explained by variation in certain factors such as sample size, distribution of clinical features, and limited generalisability. Further research focused on more participants and standardised analysis for the clinical utility of these putative biomarkers may thus be required. Furthermore, genome‐wide screening of serum lncRNAs by sequencing technologies and validation on large‐scale cohorts from multicentres are needed to better elucidate the clinical values of lncRNAs for the diagnosis of PCA [42, 43].

Conventionally, PSA is the most widely used biomarker to screen PCA. However, its low SP for cancer has raised arguments that routine use might result in overdiagnosis, overtreatment and increased healthcare cost [5, 6]. In view of the extensive application of multiple indicators for early diagnosis of cancer, great efforts have been made to identify useful biomarkers in combination with traditional PSA for increased SP and SN. Lyu et al. [44] reported improved PSA performance when combined with a panel of serum microRNAs, while Zhao et al. [14] found that simultaneous detection of PSA, MALAT1 and TMPRSS2‐ETV1 could provide a reference for assessing disease progression and predicting prognosis of PCA. In this study, we also found that the constructed ProsRISK was significantly superior to PSA alone, with improved SP and SN in stratified discriminating patients with PCA from healthy controls and benign controls.

One of the challenges in early diagnosis of PCA is the selection of the appropriate cohorts at risk, as extensive screening in the general population is not feasible [45, 46]. Current guidelines recommend screening of asymptomatic individuals with a history of familial prostate cancer and rare pathogenic mutations [47]. Additional amenable cohorts include patients with BPH [48, 49] and prostatitis [50]. All these above cohorts were recruited to validate the accuracy of the revealed biomarkers in this study. On the one hand, the threshold of ProsRISK‐Fam based on SN was used for individuals with genetic predisposition with the aim to maximise the possibilities of detecting PCA. On the other hand, the threshold of ProsRISK‐Sym focused on SP was applied to patients with symptoms suggestive of PCA. The patients with an elevated risk of PCA evaluated by ProsRISK would then need more active surveillance, including invasive and costly clinical workup. The simple serum testing with a binary ProsRISK outcome would be straightforward to perform and would likely be used as cost‐effective triaging tests.

In conclusion, our findings suggested that the established ProsRISK score based on serum lncRNAs demonstrated high accuracy in the diagnosis of PCA. Although requiring further measurement and evaluation, the PancRISK model showed great applicable potential for the stratification of patients already predisposed to develop PCA, thus enriching the population that needs clinical follow‐up.

## Author Contributions

**Xiumei Jiang:** formal analysis (equal), methodology (equal), writing – original draft (equal). **Zhongchao Liu:** formal analysis (equal), methodology (equal). **Hongxing Wang:** writing – review and editing (equal). **Lishui Wang:** conceptualization (equal), investigation (equal).

## Conflicts of Interest

The authors declare no conflicts of interest.

## Supporting information


Data S1.

## Data Availability

The data that support the findings of this study are available from the corresponding author upon reasonable request.

## References

[1] H. Sung , J. Ferlay , R. L. Siegel , et al., “Global Cancer Statistics 2020: GLOBOCAN Estimates of Incidence and Mortality Worldwide for 36 Cancers in 185 Countries,” CA: A Cancer Journal for Clinicians 71, no. 3 (2021): 209–249. 33538338 10.3322/caac.21660

[2] M. B. Culp , I. Soerjomataram , J. A. Efstathiou , F. Bray , and A. Jemal , “Recent Global Patterns in Prostate Cancer Incidence and Mortality Rates,” European Urology 77, no. 1 (2020): 38–52, 10.1016/j.eururo.2019.08.005. 31493960

[3] C. Xia , X. Dong , H. Li , et al., “Cancer Statistics in China and United States, 2022: Profiles, Trends, and Determinants,” Chinese Medical Journal 135, no. 5 (2022): 584–590. 35143424 10.1097/CM9.0000000000002108 PMC8920425

[4] T. Liu , H. Chi , J. Chen , et al., “Curcumin Suppresses Proliferation and In Vitro Invasion of Human Prostate Cancer Stem Cells by ceRNA Effect of miR‐145 and lncRNA‐ROR,” Gene 631 (2017): 29–38. 28843521 10.1016/j.gene.2017.08.008

[5] H. Van Poppel , T. Albreht , P. Basu , R. Hogenhout , S. Collen , and M. Roobol , “Serum PSA‐Based Early Detection of Prostate Cancer in Europe and Globally: Past, Present and Future,” Nature Reviews. Urology 19, no. 9 (2022): 562–572. 35974245 10.1038/s41585-022-00638-6

[6] G. Draisma , R. Etzioni , A. Tsodikov , et al., “Lead Time and Overdiagnosis in Prostate‐Specific Antigen Screening: Importance of Methods and Context,” Journal of the National Cancer Institute 101, no. 6 (2009): 374–383. 19276453 10.1093/jnci/djp001 PMC2720697

[7] J. A. Halpern , C. Oromendia , J. E. Shoag , et al., “Use of Digital Rectal Examination as an Adjunct to Prostate Specific Antigen in the Detection of Clinically Significant Prostate Cancer,” Journal of Urology 199, no. 4 (2018): 947–953. 29061540 10.1016/j.juro.2017.10.021 PMC6719551

[8] F. H. Drost , D. F. Osses , D. Nieboer , et al., “Prostate MRI, With or Without MRI‐Targeted Biopsy, and Systematic Biopsy for Detecting Prostate Cancer,” Cochrane Database of Systematic Reviews 4, no. 4 (2019): CD012663. 31022301 10.1002/14651858.CD012663.pub2 PMC6483565

[9] A. B. Herman , D. Tsitsipatis , and M. Gorospe , “Integrated lncRNA Function Upon Genomic and Epigenomic Regulation,” Molecular Cell 82, no. 12 (2022): 2252–2266. 35714586 10.1016/j.molcel.2022.05.027 PMC9219586

[10] W. X. Peng , P. Koirala , and Y. Y. Mo , “LncRNA‐Mediated Regulation of Cell Signaling in Cancer,” Oncogene 36, no. 41 (2017): 5661–5667. 28604750 10.1038/onc.2017.184 PMC6450570

[11] Q. Yang , M. Wang , J. Xu , et al., “LINC02159 Promotes Non‐Small Cell Lung Cancer Progression via ALYREF/YAP1 Signaling,” Molecular Cancer 22, no. 1 (2023): 122. 37537569 10.1186/s12943-023-01814-x PMC10401734

[12] C. Yao , L. Zeng , Q. Liu , X. Qiu , and C. Chen , “LncRNA FAM225B Regulates PDIA4‐Mediated Ovarian Cancer Cell Invasion and Migration via Modulating Transcription Factor DDX17,” Breast Journal 2023 (2023): 3970444. 37720188 10.1155/2023/3970444 PMC10501846

[13] R. Hu , P. Wu , and J. Liu , “LncRNA MAGI2‐AS3 Inhibits Prostate Cancer Progression by Targeting the miR‐142‐3p,” Hormone and Metabolic Research 54, no. 11 (2022): 754–759. 35944561 10.1055/a-1891-6864

[14] G. Zhao , Z. Pan , and P. Wang , “The Value of Combined Detection of Serum PSA, MALAT1 and TMPRSS2‐ETV1 in Evaluating the Progress and Prognosis of Prostate Cancer,” Archivos Españoles de Urología 76, no. 8 (2023): 555–562. 37960954 10.56434/j.arch.esp.urol.20237608.69

[15] S. Zhang , L. Du , L. Wang , et al., “Evaluation of Serum Exosomal LncRNA‐Based Biomarker Panel for Diagnosis and Recurrence Prediction of Bladder Cancer,” Journal of Cellular and Molecular Medicine 23, no. 2 (2019): 1396–1405. 30467945 10.1111/jcmm.14042 PMC6349164

[16] Z. Yu , C. Lu , and Y. Lai , “A Serum miRNAs Signature for Early Diagnosis of Bladder Cancer,” Annals of Medicine 55, no. 1 (2023): 736–745. 36856518 10.1080/07853890.2023.2172206 PMC9980012

[17] S. Debernardi , H. O'Brien , A. S. Algahmdi , et al., “A Combination of Urinary Biomarker Panel and PancRISK Score for Earlier Detection of Pancreatic Cancer: A Case‐Control Study,” PLoS Medicine 17, no. 12 (2020): e1003489, 10.1371/journal.pmed.1003489. 33301466 PMC7758047

[18] D. G. Altman and J. M. Bland , “Diagnostic Tests 2: Predictive Values,” BMJ 309, no. 6947 (1994): 102. 8038641 10.1136/bmj.309.6947.102 PMC2540558

[19] W. T. Wu , Y. J. Li , A. Z. Feng , et al., “Data Mining in Clinical Big Data: The Frequently Used Databases, Steps, and Methodological Models,” Military Medical Research 8, no. 1 (2021): 44. 34380547 10.1186/s40779-021-00338-z PMC8356424

[20] T. Hirose , G. Virnicchi , A. Tanigawa , et al., “NEAT1 Long Noncoding RNA Regulates Transcription via Protein Sequestration Within Subnuclear Bodies,” Molecular Biology of the Cell 25, no. 1 (2014): 169–183. 24173718 10.1091/mbc.E13-09-0558 PMC3873887

[21] M. K. Park , L. Zhang , K. W. Min , et al., “NEAT1 Is Essential for Metabolic Changes That Promote Breast Cancer Growth and Metastasis,” Cell Metabolism 33, no. 12 (2021): 2380–2397. 34879239 10.1016/j.cmet.2021.11.011 PMC8813003

[22] Y. Jia , Q. Yan , Y. Zheng , et al., “Long Non‐Coding RNA NEAT1 Mediated RPRD1B Stability Facilitates Fatty Acid Metabolism and Lymph Node Metastasis via c‐Jun/c‐Fos/SREBP1 Axis in Gastric Cancer,” Journal of Experimental & Clinical Cancer Research 41, no. 1 (2022): 287. 36171622 10.1186/s13046-022-02449-4 PMC9520879

[23] M. Taheri , E. Badrlou , B. M. Hussen , A. H. Kashi , S. Ghafouri‐Fard , and A. Baniahmad , “Importance of Long Non‐Coding RNAs in the Pathogenesis, Diagnosis, and Treatment of Prostate Cancer,” Frontiers in Oncology 13 (2023): 1123101. 37025585 10.3389/fonc.2023.1123101 PMC10070735

[24] S. Wen , Y. Wei , C. Zen , W. Xiong , Y. Niu , and Y. Zhao , “Long Non‐Coding RNA NEAT1 Promotes Bone Metastasis of Prostate Cancer Through N6‐Methyladenosine,” Molecular Cancer 19, no. 1 (2020): 171. 33308223 10.1186/s12943-020-01293-4 PMC7733260

[25] Y. Zhang , S. Pitchiaya , M. Cieślik , et al., “Analysis of the Androgen Receptor‐Regulated lncRNA Landscape Identifies a Role for ARLNC1 in Prostate Cancer Progression,” Nature Genetics 50, no. 6 (2018): 814–824. 29808028 10.1038/s41588-018-0120-1 PMC5980762

[26] J. Li , Y. Lian , C. Yan , et al., “Long Non‐Coding RNA FOXP4‐AS1 Is an Unfavourable Prognostic Factor and Regulates Proliferation and Apoptosis in Colorectal Cancer,” Cell Proliferation 50, no. 1 (2017): e12312. 27790757 10.1111/cpr.12312 PMC6529074

[27] Y. Niu , G. Wang , Y. Li , W. Guo , Y. Guo , and Z. Dong , “LncRNA FOXP4‐AS1 Promotes the Progression of Esophageal Squamous Cell Carcinoma by Interacting With MLL2/H3K4me3 to Upregulate FOXP4,” Frontiers in Oncology 12 (2022): 1041732. 36313704 10.3389/fonc.2022.1041732 PMC9616079

[28] X. Wu , Y. Xiao , Y. Zhou , Z. Zhou , and W. Yan , “LncRNA FOXP4‐AS1 Is Activated by PAX5 and Promotes the Growth of Prostate Cancer by Sequestering miR‐3184‐5p to Upregulate FOXP4,” Cell Death & Disease 10, no. 7 (2019): 472. 31209207 10.1038/s41419-019-1699-6 PMC6572815

[29] S. Ghafouri‐Fard , T. Khoshbakht , M. Taheri , and K. Ebrahimzadeh , “A Review on the Carcinogenic Roles of DSCAM‐AS1,” Frontiers in Cell and Development Biology 9 (2021): 758513. 10.3389/fcell.2021.758513 PMC8542687 34708048

[30] Y. Zhang , Y. X. Huang , D. L. Wang , et al., “LncRNA DSCAM‐AS1 Interacts With YBX1 to Promote Cancer Progression by Forming a Positive Feedback Loop That Activates FOXA1 Transcription Network,” Theranostics 10, no. 23 (2020): 10823–10837. 32929382 10.7150/thno.47830 PMC7482804

[31] V. Mugoni , Y. Ciani , C. Nardella , and F. Demichelis , “Circulating RNAs in Prostate Cancer Patients,” Cancer Letters 524 (2022): 57–69. 34656688 10.1016/j.canlet.2021.10.011

[32] M. Szilágyi , O. Pös , É. Márton , et al., “Circulating Cell‐Free Nucleic Acids: Main Characteristics and Clinical Application,” International Journal of Molecular Sciences 21, no. 18 (2020): 6827. 32957662 10.3390/ijms21186827 PMC7555669

[33] P. Qi , X. Y. Zhou , and X. Du , “Circulating Long Non‐Coding RNAs in Cancer: Current Status and Future Perspectives,” Molecular Cancer 15, no. 1 (2016): 39. 27189224 10.1186/s12943-016-0524-4 PMC4869386

[34] Y. Xie , Y. Zhang , L. Du , et al., “Circulating Long Noncoding RNA Act as Potential Novel Biomarkers for Diagnosis and Prognosis of Non‐Small Cell Lung Cancer,” Molecular Oncology 12, no. 5 (2018): 648–658. 29504701 10.1002/1878-0261.12188 PMC5928376

[35] R. Wang , L. Du , X. Yang , et al., “Identification of Long Noncoding RNAs as Potential Novel Diagnosis and Prognosis Biomarkers in Colorectal Cancer,” Journal of Cancer Research and Clinical Oncology 142, no. 11 (2016): 2291–2301. 27591862 10.1007/s00432-016-2238-9 PMC11819400

[36] Y. Gao , J. W. Wang , J. Y. Ren , et al., “Long Noncoding RNAs in Gastric Cancer: From Molecular Dissection to Clinical Application,” World Journal of Gastroenterology 26, no. 24 (2020): 3401–3412. 32655264 10.3748/wjg.v26.i24.3401 PMC7327794

[37] C. H. C. Sukowati , L. K. D. Cabral , C. Tiribelli , and D. Pascut , “Circulating Long and Circular Noncoding RNA as Non‐Invasive Diagnostic Tools of Hepatocellular Carcinoma,” Biomedicine 9, no. 1 (2021): 90. 10.3390/biomedicines9010090 PMC7832835 33477833

[38] Y. Wang , D. Zhang , C. Zhang , and Y. Sun , “The Diagnostic and Prognostic Value of Serum lncRNA NEAT1 in Colorectal Cancer,” Cancer Management and Research 12 (2020): 10985–10992, 10.2147/CMAR.S269978. 33173332 PMC7646461

[39] M. Mohyeldeen , S. Ibrahim , O. Shaker , and H. Helmy , “Serum Expression and Diagnostic Potential of Long Non‐Coding RNAs NEAT1 and TUG1 in Viral Hepatitis C and Viral Hepatitis C‐Associated Hepatocellular Carcinoma,” Clinical Biochemistry 84 (2020): 38–44. 32526227 10.1016/j.clinbiochem.2020.06.005

[40] A. A. A. El‐Fattah , N. A. H. Sadik , O. G. Shaker , A. Mohamed Kamal , and N. N. Shahin , “Serum Long Non‐Coding RNAs PVT1, HOTAIR, and NEAT1 as Potential Biomarkers in Egyptian Women With Breast Cancer,” Biomolecules 11, no. 2 (2021): 301. 33670447 10.3390/biom11020301 PMC7922136

[41] X. Mao , G. Shaw , S. Y. James , et al., “Detection of TMPRSS2:ERG Fusion Gene in Circulating Prostate Cancer Cells,” Asian Journal of Andrology 10, no. 3 (2008): 467–473. 18385909 10.1111/j.1745-7262.2008.00401.x

[42] W. Zheng , C. Chen , S. Chen , C. Fan , and H. Ruan , “Integrated Analysis of Long Non‐Coding RNAs and mRNAs Associated With Peritendinous Fibrosis,” Journal of Advanced Research 15 (2018): 49–58. 30581612 10.1016/j.jare.2018.08.001 PMC6300459

[43] J. Sun , R. Jiang , M. Song , et al., “Pathological Grade‐Associated Transcriptome Profiling of lncRNAs and mRNAs in Gliomas,” Frontiers in Oncology 10 (2020): 253. 32211318 10.3389/fonc.2020.00253 PMC7076085

[44] J. Lyu , L. Zhao , F. Wang , et al., “Discovery and Validation of Serum microRNAs as Early Diagnostic Biomarkers for Prostate Cancer in Chinese Population,” BioMed Research International 2019 (2019): 9306803. 31534967 10.1155/2019/9306803 PMC6732591

[45] J. McHugh , E. J. Saunders , T. Dadaev , et al., “Prostate Cancer Risk in Men of Differing Genetic Ancestry and Approaches to Disease Screening and Management in These Groups,” British Journal of Cancer 126, no. 10 (2022): 1366–1373. 34923574 10.1038/s41416-021-01669-3 PMC9090767

[46] C. Arsov , P. Albers , K. Herkommer , et al., “A Randomized Trial of Risk‐Adapted Screening for Prostate Cancer in Young Men‐Results of the First Screening Round of the PROBASE Trial,” International Journal of Cancer 150, no. 11 (2022): 1861–1869. 35076933 10.1002/ijc.33940

[47] T. M. Seibert , I. P. Garraway , A. Plym , et al., “Genetic Risk Prediction for Prostate Cancer: Implications for Early Detection and Prevention,” European Urology 83, no. 3 (2023): 241–248. 36609003 10.1016/j.eururo.2022.12.021

[48] D. D. Ørsted and S. E. Bojesen , “The Link Between Benign Prostatic Hyperplasia and Prostate Cancer,” Nature Reviews. Urology 10, no. 1 (2013): 49–54. 23165396 10.1038/nrurol.2012.192

[49] C. De Nunzio , G. Kramer , M. Marberger , et al., “The Controversial Relationship Between Benign Prostatic Hyperplasia and Prostate Cancer: The Role of Inflammation,” European Urology 60, no. 1 (2011): 106–117. 21497433 10.1016/j.eururo.2011.03.055

[50] S. Doat , M. Marous , X. Rebillard , et al., “Prostatitis, Other Genitourinary Infections and Prostate Cancer Risk: Influence of Non‐Steroidal Anti‐Inflammatory Drugs? Results From the EPICAP Study,” International Journal of Cancer 143, no. 7 (2018): 1644–1651. 29696626 10.1002/ijc.31565

