# Supplementary material for: Discovery of lncRNA‐Based ProsRISK Score in Serum as Potential Biomarkers for Improved Accuracy of Prostate Cancer Detection
Source: J Cell Mol Med. 2025 Apr 21;29(8):e70555. doi: 10.1111/jcmm.70555 (PMC12011553; doi:10.1111/jcmm.70555)
Supplement: Supplementary file 1 — Data S1. [file JCMM-29-e70555-s001.docx]

**Supplementary Figures**


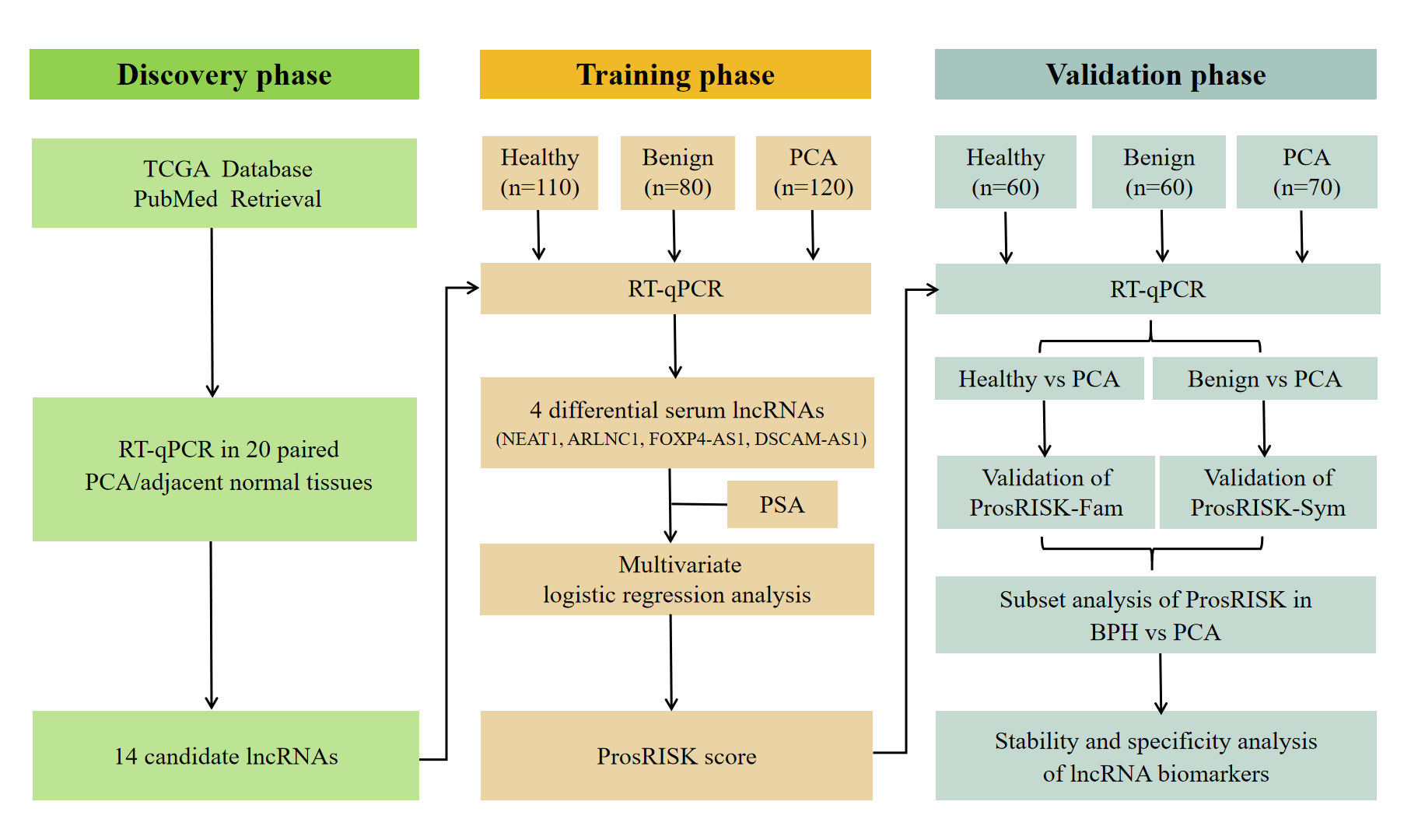


FIGURE S1 The flowchart of our study design.


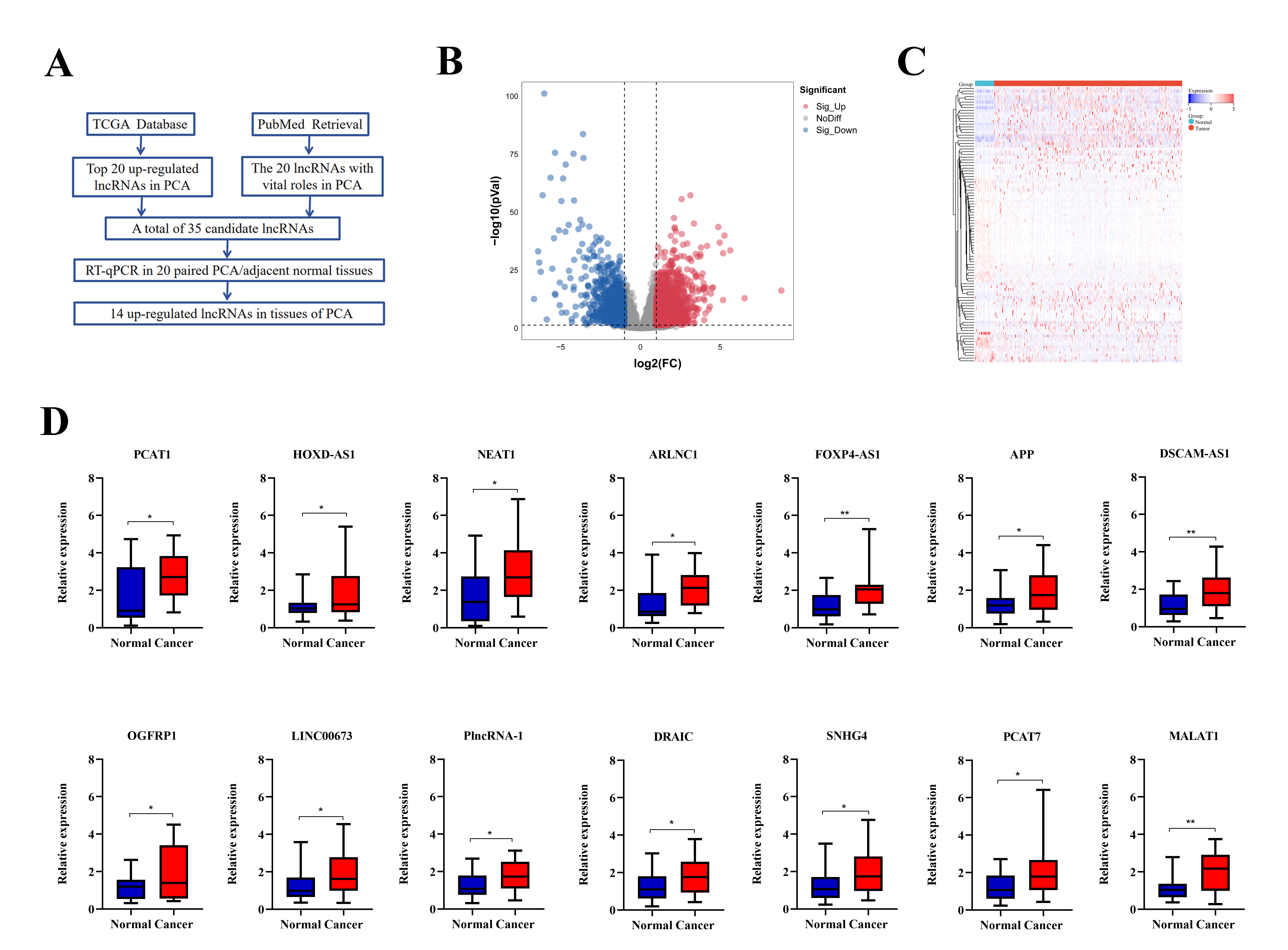


FIGURE S2. The selection of 14 up-regulated lncRNAs in PCA in the discovery phase. (A) The overview of selection criteria based on TCGA database, literature review and validation in tissues of PCA. (B) Volcano plot of expression changes larger than twice of lncRNAs in PCA and normal tissues from TCGA database (criteria: p<0.05 and |log2FC|≥1). (C) Heatmap of lncRNAs in PCA samples from TCGA database. (D) Differential expression of 14 lncRNAs in 20 paired PCA/adjacent normal tissues revealed by RT-qPCR assays, ***p*<0.01, **p*<0.05.


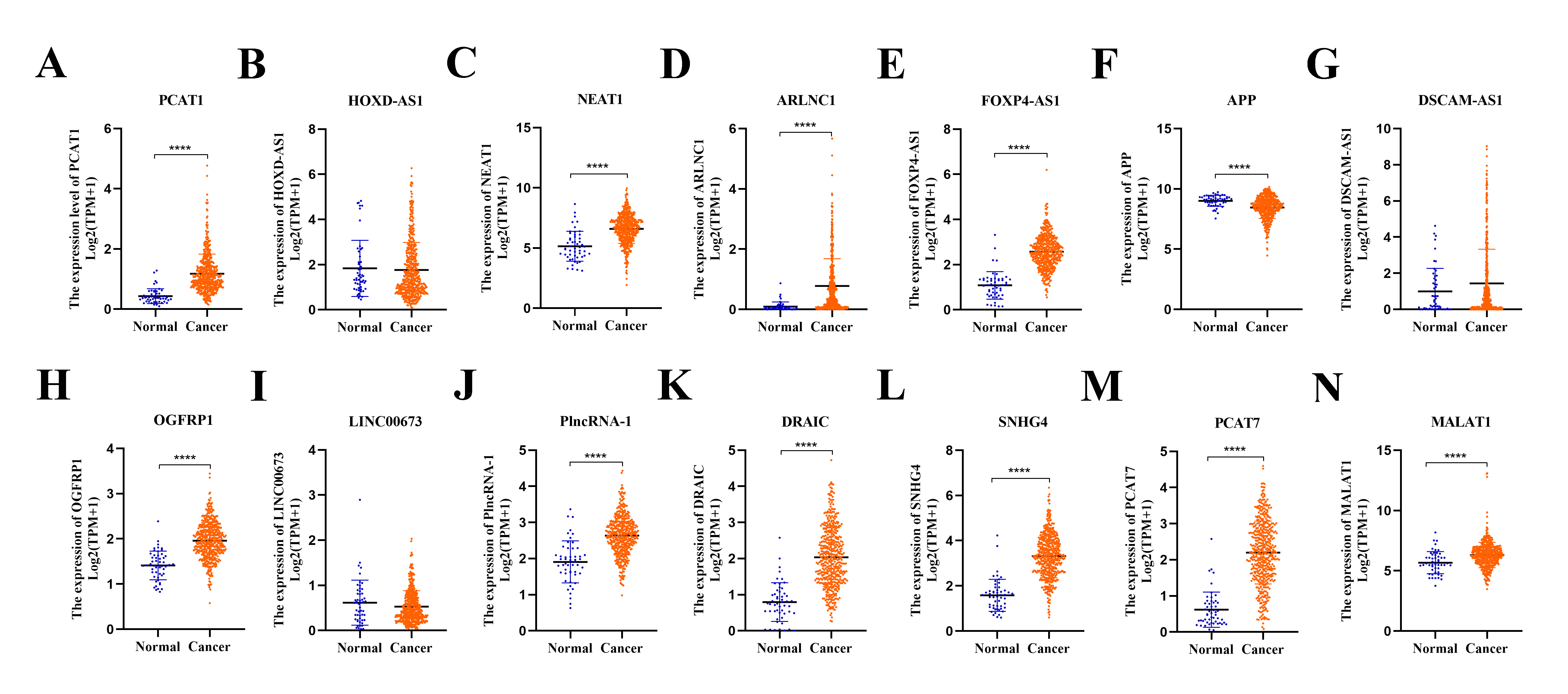


FIGURE S3. Expression levels of 14 lncRNAs in PCA revealed by TCGA database, *****p*<0.0001.


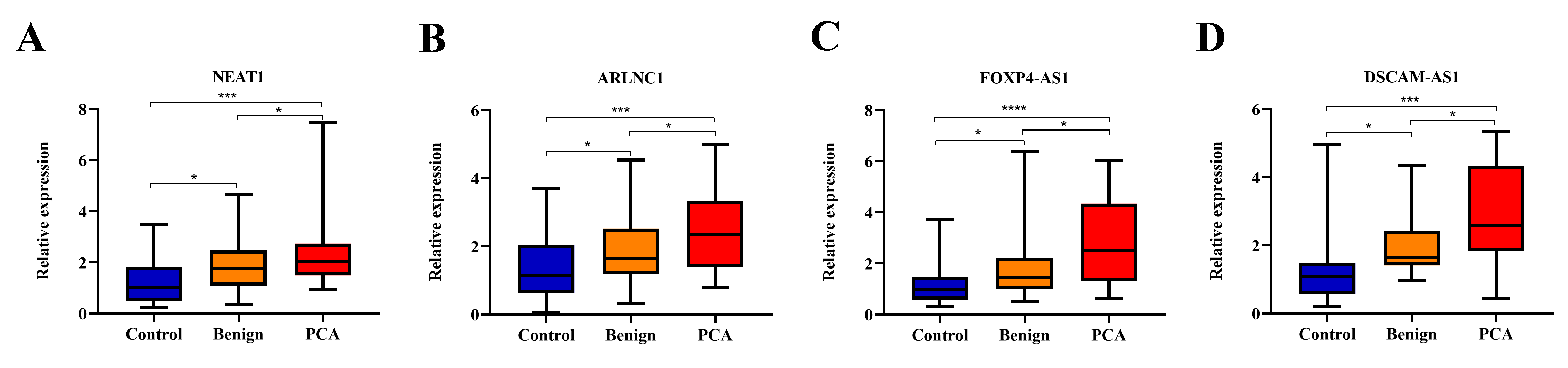


FIGURE S4 Differential expression levels of NEAT1, ARLNC1, FOXP4-AS1, DSCAM-AS1 in serum from patients with PCA (n=30), benign controls (n=30) and healthy controls (n=30) using RT-qPCR assays, *****p*<0.0001, ****p*<0.001, **p*<0.05.


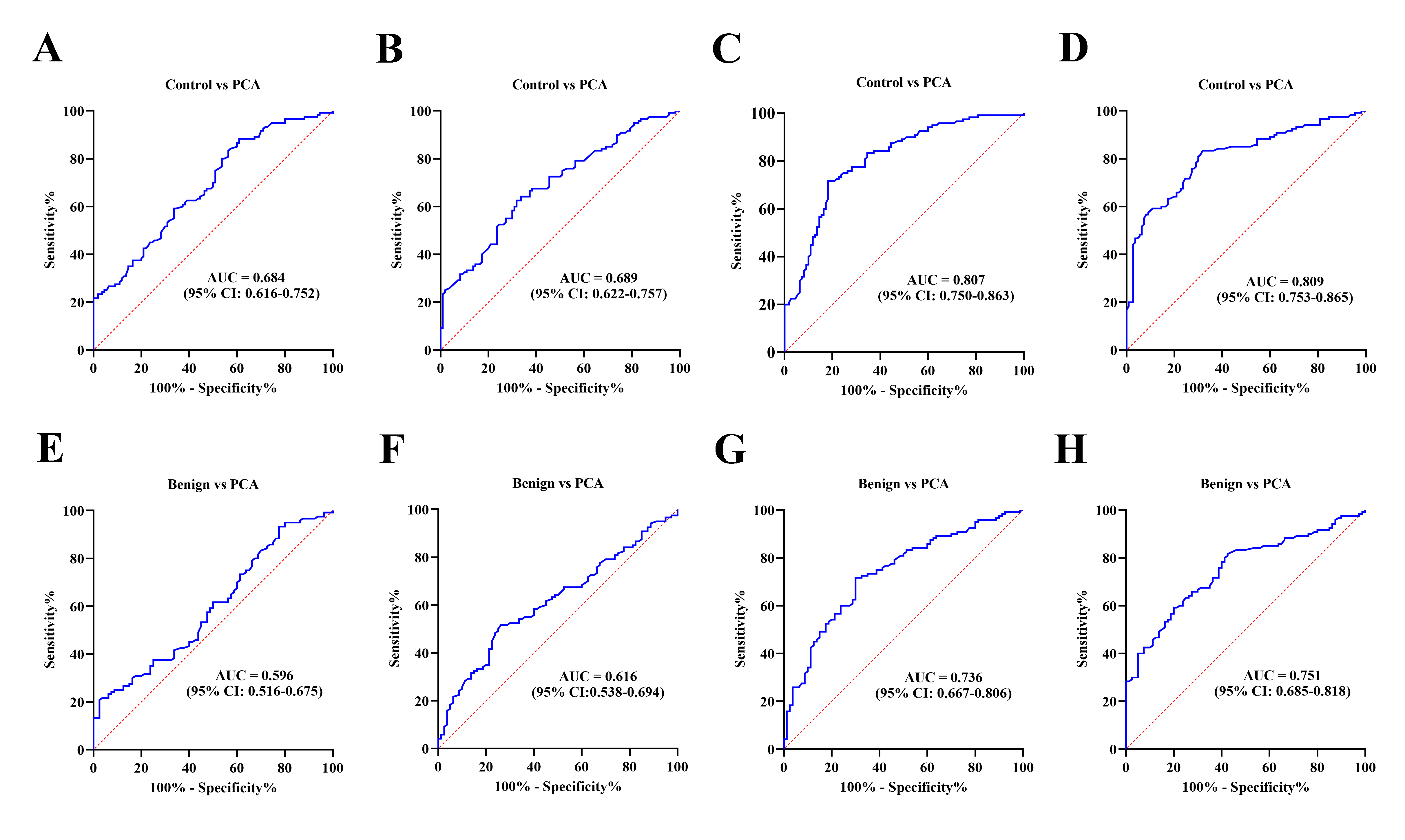


FIGURE S5 ROC curve analysis for the detection of PCA from healthy controls using NEAT1(A), ARLNC1(B), FOXP4-AS1(C), DSCAM-AS1(D) and for the detection of PCA from benign controls using NEAT1(E), ARLNC1(F), FOXP4-AS1(G), DSCAM-AS1(H) in the training set.


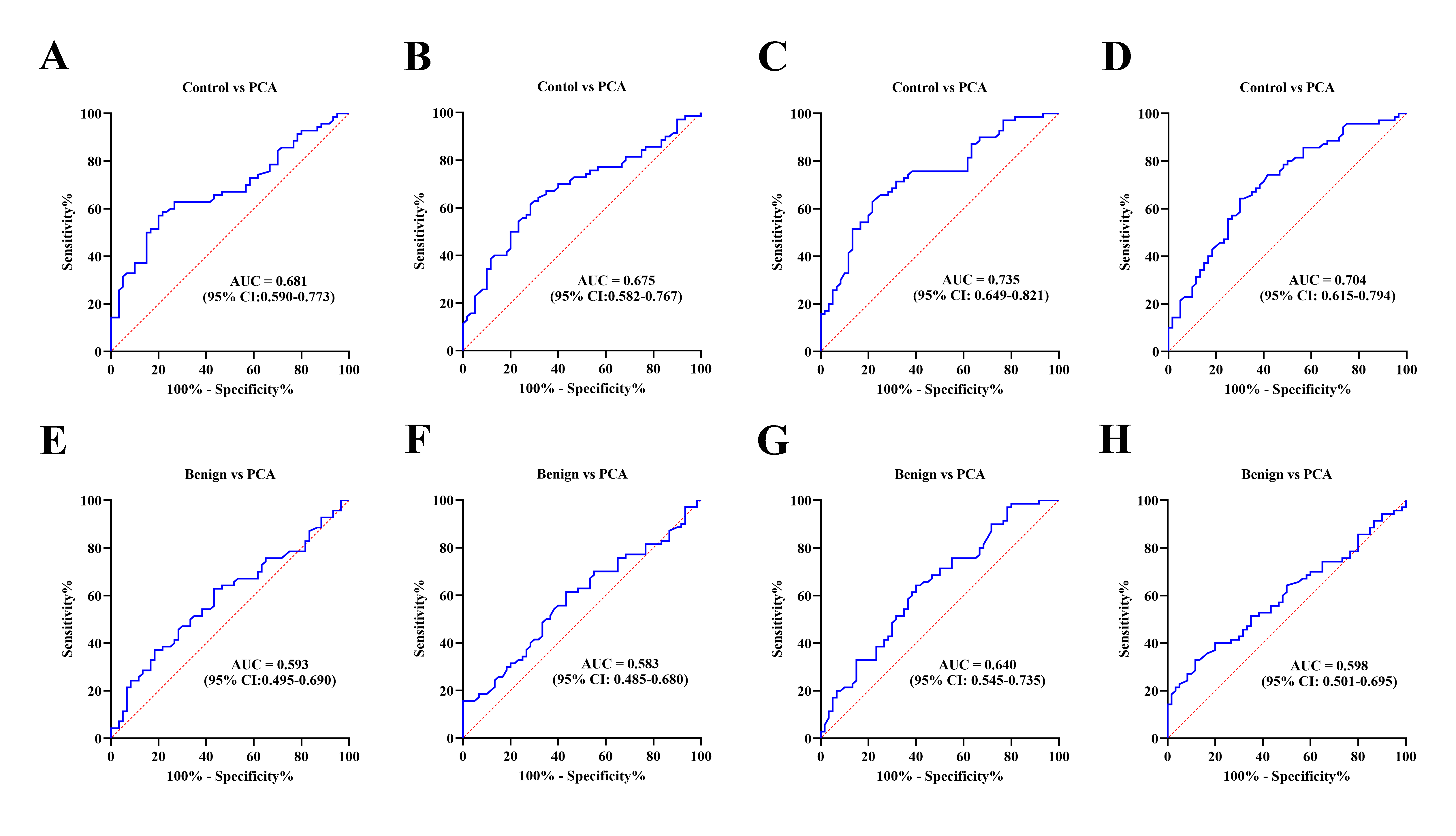


FIGURE S6 ROC curve analysis for the detection of PCA from healthy controls using NEAT1(A), ARLNC1(B), FOXP4-AS1(C), DSCAM-AS1(D) and for the detection of PCA from benign controls using NEAT1(E), ARLNC1(F), FOXP4-AS1(G), DSCAM-AS1(H) in the validation set.


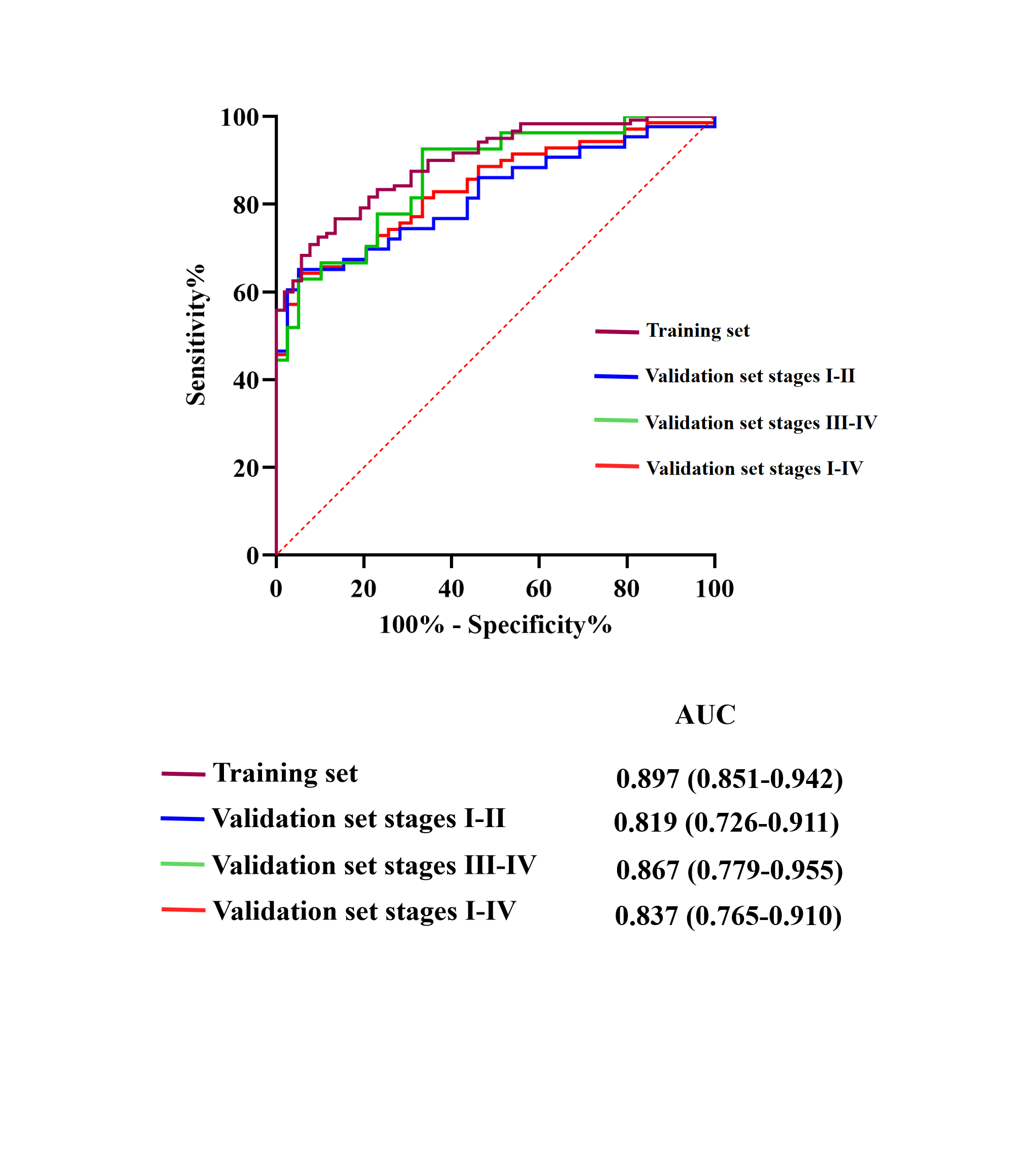


FIGURE S7 ROC curve analysis for the ProsRISK in discriminating PCA from BPH in the training set and validation set.


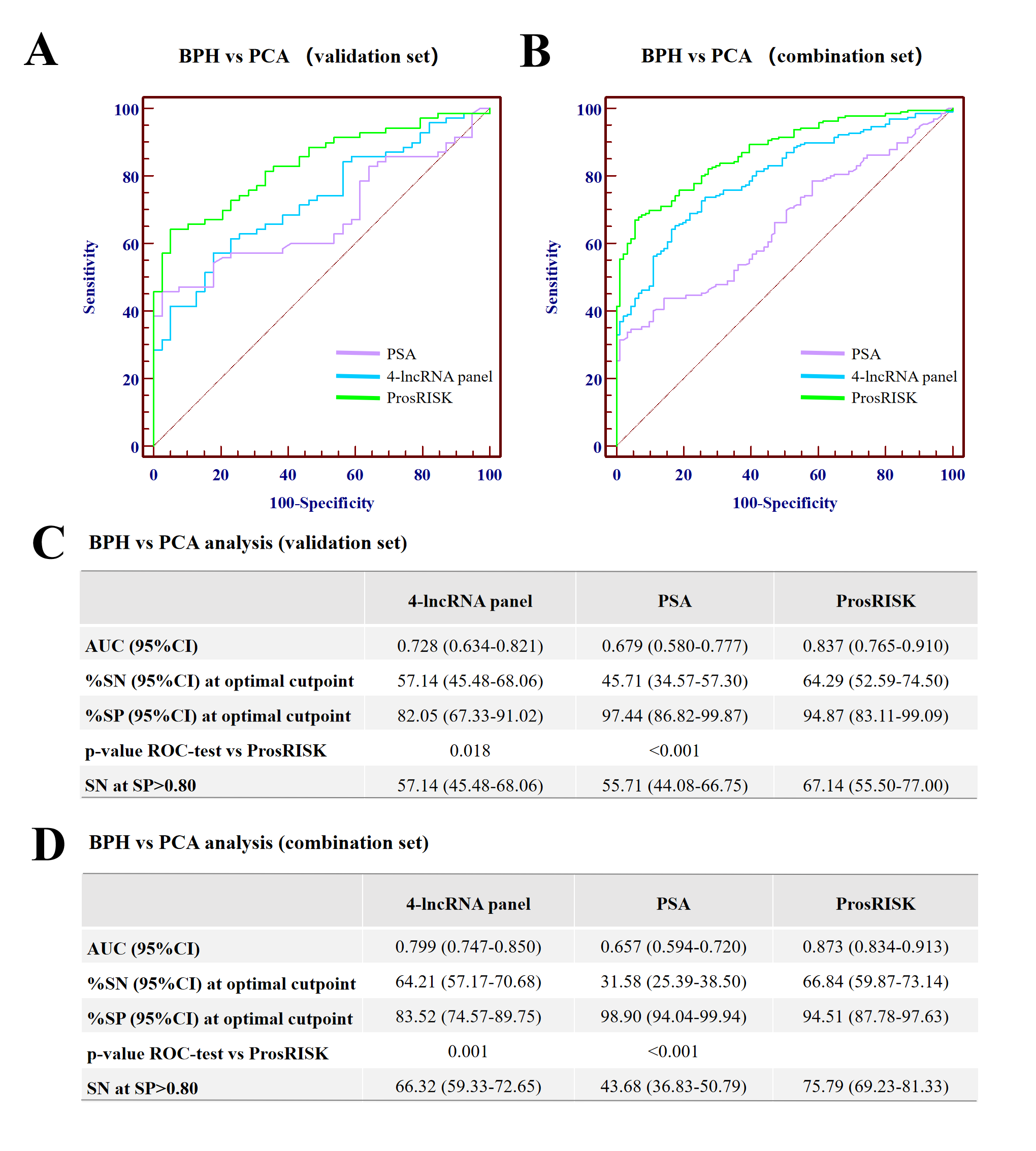


FIGURE S8 Performance analysis of the ProsRISK for diagnosis of PCA from BPH in the validation set (A and C) and combination set (B and D).


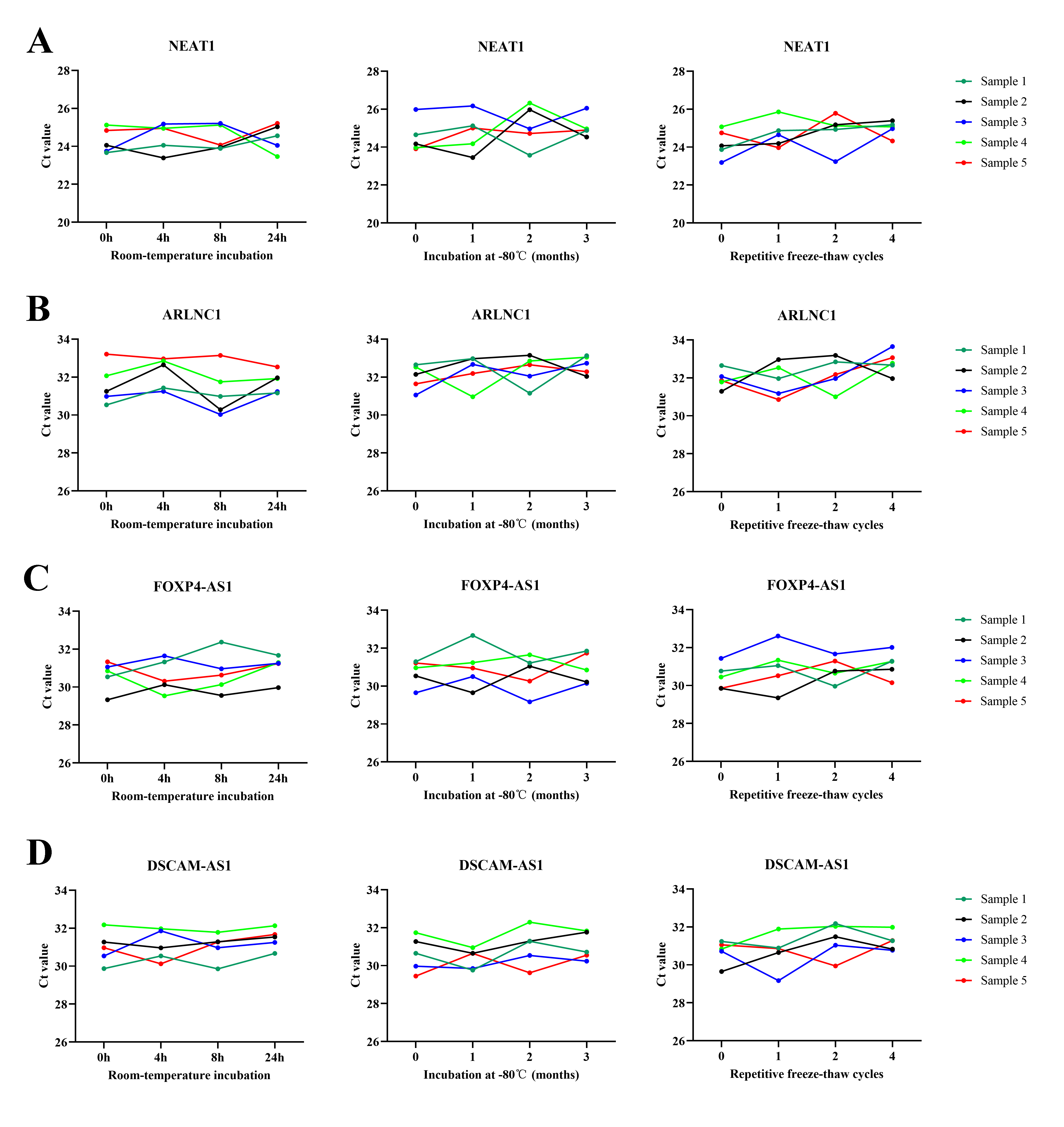


FIGURE S9 Stability analysis of lncRNA expression in serum. Prolonged incubation at room-temperature, prolonged incubation at –80℃, or repetitive freeze-thaw cycles had no effects on the expression levels of NEAT1(A), ARLNC1(B), FOXP4-AS1(C), and DSCAM-AS1(D) in serum.

**Supplementary Tables**

**TABLE S1** Characteristics of study participants in the training and validation sets.

| **Variable, N. (%)** | **Training phase** | | |  | **Validation phase** | | |
| --- | --- | --- | --- | --- | --- | --- | --- |
|  | Healthy controls | Benign controls | PCA |  | Healthy controls | Benign controls | PCA |
| **Age (years)** | | | | | | | |
| <65 | 42 (38.2) | 35 (43.7) | 47 (39.2) |  | 21 (35) | 30 (50) | 27 (38.6) |
| ≥65 | 68 (61.8) | 45 (56.3) | 73 (60.8) |  | 39 (65) | 30 (50) | 43 (61.4) |
| **Other prostate conditions** | | | | | | | |
| BPH | - | 52 (65) | - |  | - | 39 (65) | - |
| Prostatitis | - | 28 (35) | - |  | - | 21 (35) | - |
| **Preoperation PSA level (ng/mL)** | | | | | | | |
| ≤20 | 110 (100) | 80 (100) | 88 (73.3) |  | 59 (98.3) | 53 (88.3) | 37 (52.9) |
| >20 | 0 (0) | 0(0) | 32 (26.7) |  | 1 (1.7) | 7 (11.7) | 33 (47.1) |
| **Gleason score** | | | | | | | |
| ≤7 | - | - | 72 (60) |  | - | - | 39 (55.7) |
| >7 | - | - | 48 (40) |  | - | - | 31 (44.3) |
| **Stage** |  | - |  |  |  |  |  |
| I–II | - | - | 82 (68.3) |  | - | - | 43 (61.4) |
| III–IV | - | - | 38 (31.7) |  | - | - | 27 (38.6) |
| **Lymph node metastasis** | | | | | | | |
| Negative | - | - | 99 (82.5) |  | - | - | 60 (85.7) |
| Positive | - | - | 21 (17.5) |  | - | - | 10 (14.3) |
| **Bone metastasis** | | | | | | | |
| Negative | - | - | 111 (92.5) |  | - | - | 65 (92.9) |
| Positive | - | - | 9 (7.5) |  | - | - | 5 (7.1) |

**TABLE S2** Primers of 4 differentially expressed lncRNAs and GAPDH used for RT-qPCR.

| **Gene** | **Primer** | **Sequencing** |
| --- | --- | --- |
| NEAT1 | Forward (5’-3’) | GTGTGATCTGAAAACCCTGCT |
|  | Reverse (5’-3’) | CCCAGAAGACAGAAAGATCCCA |
| ARLNC1 | Forward (5’-3’) | CCTTGTCCACTGGAACTCGT |
|  | Reverse (5’-3’) | TATAACCTTGGGGGCCATGA |
| FOXP4-AS1 | Forward (5’-3’) | ATGGAAAAGGGGCTGACTGG |
|  | Reverse (5’-3’) | CTCTGGGAATGGGGTGTACG |
| DSCAM-AS1 | Forward (5’-3’) | ACAGAGATGGACGACGGATC |
|  | Reverse (5’-3’) | TTGTGAGCCTGAGAGATCCC |
| GAPDH | Forward (5’-3’) | GCACCGTCAAGGCTGAGAAC |
|  | Reverse (5’-3’) | TGGTGAAGACGCCAGTGGA |

**TABLE S3** The top 20 up-regulated lncRNAs in PCA revealed by TCGA database.

| **LncRNA** | **Log_2_FC(Cancer/Normal)** | ***p* value** |
| --- | --- | --- |
| HPN-AS1 | 3.13288347166291 | 8.9348298486606E-61 |
| PCAT7 | 2.58438562777644 | 4.06516768772197E-59 |
| AL031123.2 | 2.10439713555398 | 8.54433772007322E-51 |
| PCSEAT | 3.35977047022402 | 2.12035447200094E-48 |
| AL133325.3 | 4.88560818556893 | 7.30723586170837E-47 |
| SNHG4 | 2.13975172821867 | 1.17182359717809E-46 |
| FOXP4-AS1 | 2.15859051755505 | 3.74638975282964E-46 |
| DRAIC | 2.22543847194189 | 6.6353060167308E-45 |
| CTBP1-AS | 2.59476743229231 | 1.01870026778935E-43 |
| LINC01475 | 5.27007659597783 | 4.49399310665094E-43 |
| VPS9D1-AS1 | 1.90848658580852 | 5.96359965470357E-43 |
| LINC01509 | 2.16552921541679 | 1.31725759747532E-40 |
| AL353637.2 | 4.9755848981118 | 6.43647547309213E-40 |
| B3GAT1-DT | 2.93964697858737 | 1.58262951173739E-39 |
| AC009119.1 | 2.67410087940681 | 5.79834725133415E-39 |
| ARLNC1 | 4.18265467325578 | 1.90374208390504E-38 |
| AL031985.3 | 1.10703849426764 | 6.86163525166322E-38 |
| AC012531.1 | 3.79756546196374 | 9.71038789184123E-38 |
| PCAT1 | 2.14436533745685 | 1.47712485629507E-36 |
| AL353622.1 | 2.04651384158469 | 1.61463807601928E-36 |

**Table S4** An overview of 20 most relevant lncRNAs with PCA revealed by PubMed.

| **LncRNA** | **Function** | **Mechanism** | **Authors, year** | **Refs (PMID)** |
| --- | --- | --- | --- | --- |
| PCAT1 | Promotes progression and induces castration-resistance | Activates AKT and NF-κB signaling by regulating the PHLPP/FKBP51/IKKα complex | Shang *et al*, 2019 | 30773595 |
|  | Inhibits ferroptosis and enhances docetaxel-resistance | Increases c-Myc stability and promotes SLC7A11 expression by competing for miR-25-3p | Jiang *et al*, 2022 | 35402284 |
|  | Promotes proliferation and cell cycle | Mediates the expression of CENPF, ID1, and ID3 | Yang *et al*, 2019 | 31090454 |
|  |  | Interacts with AR/LSD1 and is required for their recruitment to enhance GNMT/DHCR24 | Guo *et al*, 2016 | 27526323 |
|  | Promotes proliferation and inhibits apoptosis | Acts as an oncogene and genetic variant in PCAT1 contributes to the susceptibility to PCA | Yuan *et al*, 2018 | 29721051 |
|  |  | Regulates FSCN1 via miR-145-5p | Xu *et al*, 2017 | 28922730 |
| HOXD-AS1 | Promotes metastasis | Acts as competing endogenous RNA to modulate the miR-361-5p/FOXM1 | Jiang *et al*, 2021 | 34864822 |
|  | Promotes proliferation and chemo-resistance | Recruits WDR5 to directly regulate the expression of target genes by mediating H3K4me3 | Gu *et al*, 2017 | 28487115 |
| NEAT1 | Promotes proliferation | Regulates LDHA expression and inhibits the secretion of CD8^+^ T-lymphocyte factors | Xia *et al*, 2022 | 35263995 |
|  |  | Binds to miR-98-5p and promotes expression of oncogene HMGA2 | Guo *et al*, 2019 | 31481527 |
|  |  | Promotes the transcriptional activity of CDC5L | Li *et al*, 2018 | 29871935 |
|  |  | Modulates the SRC3/IGF1R/AKT signaling pathway | Xiong *et al*, 2018 | 29225160 |
|  | Promotes bone metastasis | Upregulates RUNX2 by binding to miR-205-5p via the SFPQ/PTBP2 axis | Mo *et al*, 2021 | 34459124 |
|  |  | Acts through N6-methyladenosine and serves as a bridge to facility the binding between CYCLINL1 and CDK19 and promoted the Pol II ser2 phosphorylation | Wen *et al*, 2020 | 33308223 |
|  | Promotes docetaxel resistance | Regulates ACSL4 via sponging miR-34a-5p and miR-204-5p | Jiang *et al*, 2020 | 31672604 |
|  | Promotes progression | Serves as ERα-regulated intergenic lncRNAs and drives oncogenic growth | Chakravarty *et al*, 2014 | 25415230 |
| ARLNC1 | Promotes progression | Is induced by AR protein and promotes AR stabilization via RNA-RNA interaction and increases AR expression, global AR signaling and cancer cell growth | Zhang *et al*, 2018 | 29808028 |
| FOXP4-AS1 | Promotes proliferation | Is activated by SP4 and functions as a ceRNA of miR-3130-3p | Gu *et al*, 2022 | 37114256 |
|  |  | Is activated by PAX5 and sponges miR-3184-5p to up-regulate FOXP4 | Wu *et al*, 2019 | 31209207 |
| APP | Promotes proliferation and invasion | Competitively binds miR-218 to facilitate ZEB2/CDH2 expression | Shi *et al*, 2020 | 31107971 |
| OGFRP1 | Promotes progression | Functions as a ceRNA by regulating SARM1 level via miR-124-3p | Yan *et al*, 2020 | 32428870 |
|  | Promotes chemo-resistance | Binds and sequesters miR-149-5p, thereby indirectly regulating IL-6 expression | Wang *et al*, 2021 | 34293716 |
| LINC00673 | Promotes proliferation and drug resistance | Depresses demethylation of KLF4 gene promoter | Jiang *et al*, 2020 | 31881124 |
| DSCAM-AS1 | Promotes progression | Serves as lineage-specific oncogenic lncRNA regulated by FOXA1 | Zhang *et al*, 2020 | 32929382 |
| PlncRNA-1 | Promotes progression | Decreases PTEN/phosphorylated PTEN and increases Akt /phosphorylated Akt | Cui *et al*, 2021 | 33848262 |
|  |  | Sponges AR-targeting miRNAs to protect AR from miRNA-mediated down-regulation | Fang *et al*, 2016 | 26808578 |
|  | Promotes proliferation and inhibits apoptosis | Increases androgen receptor (AR) mRNA, protein and AR downstream target | Cui *et al*, 2013 | 22264502 |
| PCAT7 | Promotes bone metastasis | Activates TGF-β/SMAD signaling by upregulating TGFBR1 via sponging miR-324-5p | Lang *et al*, 2020 | 31925912 |
| MALAT1 | Promotes proliferation | Acts through METTL3-mediated m6A modification by activating PI3K/AKT signaling | Mao *et al*, 2022 | 36073002 |
|  |  | Sponges miR-145 and upregulates TGF-β1-induced EMT via SMAD3 and TGFBR2 | Zhang *et al*, 2021 | 34295718 |
|  | Promotes progression | Increases the levels of transcription factor STAT3 and E2F1 | Jing *et al*, 2022 | 35085770 |
|  |  | Increases BIRC mRNA and protein expression through upregulating miR-140 | Hao *et al*, 2020 | 31935634 |
|  | Promotes migration and invasion | Competes with CORO1C for the binding sites of miR-1-3p | Dai *et al*, 2019 | 31485645 |
| SNHG4 | Promotes progression | Functions as a ceRNA for miR-377 through regulation of ZIC5 | Wang *et al*, 2020 | 31608997 |
| lncAMPC | Promotes metastasis and immunosuppression | Upregulates LIF by sponging miR-637 and induces LIFR to stimulate Jak1-STAT3 pathway | Zhang *et al*, 2020 | 32592689 |
| TTTY15 | Promotes progression | Sponges let-7 consequently increasing CDK6 and FN1 expression | Xiao *et al*, 2019 | 30527798 |
| HULLK | Promotes proliferation | Is dramatically upregulated by androgen in a dose-dependent manner, and the anti-androgen enzalutamide completely blocked this hormone-induced increase | Ta *et al*, 2019 | 31253147 |
| CCAT1 | Promotes proliferation | Sponges miR-28-5p and Acts as a scaffold for DDX5 and AR transcriptional complex | You *et al*, 2019 | 31387890 |
|  | Promotes migration, and invasion | Negatively regulates miR-490-3p expression and subsequently regulates FRAT1 expression | Cai *et al*, 2021 | 34319909 |
|  |  | Decreases the M2 macrophages polarization by down-regulating miR-148a | Liu *et al*, 2019 | 30221381 |
|  | Enhances sensitivity of paclitaxel | Regulates miR-24-3p and FSCN1 | Li *et al*, 2020 | 32089062 |
| SCHLAP1 | Promotes proliferation and metastasis | Interacts with EZH2 to mediate promoter methylation modification of multiple miRNAs | Huang *et al*, 2021 | 33589600 |
|  |  | Targets miR-198 and promotes the MAPK1 pathway | Li *et al*, 2018 | 28492138 |
|  | Promotes progression | Antagonizes the genome-wide localization and regulatory functions of SWI/SNF chromatin-modifying complex | Prensner *et al*, 2013 | 24076601 |
| LINC00115 | Promotes proliferation and invasion | Serves as a ceRNA through sponging miR-212-5p to release FZD5 expression and promotes the Wnt/β-catenin signalling pathway | Peng *et al*, 2021 | 34697900 |
| H19 | Promotes invasion and treatment resistance | Facilitates the PRC2 complex in regulating methylation changes at H3K27me3/H3K4me3 histone sites of AR-driven and NEPC-related genes and induces alterations in genome-wide DNA methylation on CpG sites | Singh *et al*, 2021 | 34934057 |
|  | Promotes metastasis | Regulates β3 and β4 integrins upon estrogen and hypoxia | Bacci *et al*, 2019 | 31426484 |

**TABLE S5** Expression levels of 35 lncRNAs by RT-qPCR assays in tissues of PCA.

| **LncRNA** | **Normal** | **PCA** | ***p*** |  | **LncRNA** | **Normal** | **PCA** | ***p*** |
| --- | --- | --- | --- | --- | --- | --- | --- | --- |
| HPN-AS1 | 0.223-3.877 | 0.431-3.931 | 0.413 |  | PCAT1 | 0.116-4.739 | 0.815-4.940 | 0.0123 |
| AL353622.1 | - | - | - |  | HOXD-AS1 | 0.322-2.855 | 0.383-5.402 | 0.0425 |
| AL031123.2 | - | - | - |  | NEAT1 | 0.0988-4.926 | 0.591-6.871 | 0.0115 |
| PCSEAT | 0.428-2.227 | 0.597-3.668 | 0.694 |  | APP | 0.193-3.080 | 0.326-4.417 | 0.0387 |
| AL133325.3 | - | - | - |  | OGFRP1 | 0.319-2.626 | 0.418-4.510 | 0.0443 |
| SNHG4 | 0.235-3.487 | 0.461-4.763 | 0.0326 |  | LINC00673 | 0.354-3.588 | 0.332-4.541 | 0.0462 |
| FOXP4-AS1 | 0.179-2.668 | 0.715-5.262 | 0.0071 |  | PlncRNA-1 | 0.314-2.694 | 0.457-3.116 | 0.0249 |
| DRAIC | 0.197-3.028 | 0.417-3.780 | 0.0469 |  | PCAT7 | 0.234-2.719 | 0.433-6.422 | 0.0202 |
| CTBP1-AS | 0.068-5.661 | 0.218-3.509 | 0.412 |  | MALAT1 | 0.365-2.784 | 0.275-3.750 | 0.0047 |
| LINC01475 | 0.255-4.857 | 0.432-5.816 | 0.088 |  | lncAMPC | 0.102-3.237 | 0.115-2.150 | 0.0595 |
| VPS9D1-AS1 | - | - | - |  | TTTY15 | 0.226-5.712 | 0.488-3.191 | 0.516 |
| LINC01509 | - | - | - |  | HULLK | 0.298-4.446 | 0.254-3.277 | 0.351 |
| AL353637.2 | - | - | - |  | CCAT1 | 0.159-4.662 | 0.3792-3.162 | 0.570 |
| B3GAT1-DT | - | - | - |  | SCHLAP1 | 0.272-5.698 | 0.344-3.459 | 0.457 |
| AC009119.1 | - | - | - |  | LINC00115 | 0.290-2.321 | 0.505-2.779 | 0.165 |
| ARLNC1 | 0.262-3.907 | 0.782-3.989 | 0.0116 |  | H19 | 0.141-4.533 | 0.207-4.348 | 0.280 |
| AL031985.3 | - | - | - |  | DSCAM-AS1 | 0.291-2.443 | 0.466-4.283 | 0.005 |
| AC012531.1 | 0.472-2.229 | 0.535-3.286 | 0.130 |  |  |  |  |  |

Data were presented as (Minimum to Maximum).

**TABLE S6** Performance of the ProsRISK with different cutoffs: specificity at fixed sensitivity in the validation set.

| **Sensitivity**  **cutoff** | **Specificity** | | |
| --- | --- | --- | --- |
|  | **Control versus PCA Ⅰ-Ⅱ** | **Control versus PCA Ⅲ-Ⅳ** | **Control versus PCA Ⅰ-Ⅳ** |
| **0.80** | 0.783 (0.664-0.869) | 0.917 (0.819-0.964) | 0.900 (0.799-0.953) |
| **0.85** | 0.783 (0.664-0.869) | 0.900 (0.799-0.953) | 0.783 (0.664-0.869) |
| **0.90** | 0.683 (0.558-0.787) | 0.883 (0.778-0.942) | 0.783 (0.664-0.869) |
| **0.95** | 0.583 (0.457-0.699) | 0.783 (0.664-0.869) | 0.633 (0.507-0.744) |

**TABLE S7** Performance of the ProsRISK with different cutoffs: sensitivity at fixed specificity in the validation set.

| **Specificity**  **cutoff** | **Sensitivity** | | |
| --- | --- | --- | --- |
|  | **Benign versus PCA Ⅰ-Ⅱ** | **Benign versus PCA Ⅲ-Ⅳ** | **Benign versus PCA Ⅰ-Ⅳ** |
| **0.80** | 0.698 (0.549-0.814) | 0.704 (0.515-0.842) | 0.700 (0.585-0.795) |
| **0.85** | 0.651 (0.502-0.776) | 0.667 (0.478-0.814) | 0.657 (0.540-0.758) |
| **0.90** | 0.628 (0.479-0.756) | 0.630 (0.442-0.785) | 0.629 (0.512-0.732) |
| **0.95** | 0.605 (0.456-0.736) | 0.556 (0.373-0.742) | 0.586 (0.469-0.694) |

**TABLE S8** Positive and negative predictive values of the ProsRISK for Control vs PCA in the validation set.

| **Prevalence** | **Control vs PCA I-II** | | | | **Control vs PCA Ⅲ-Ⅳ** | | | |
| --- | --- | --- | --- | --- | --- | --- | --- | --- |
|  | **ProsRISK** | | | | **ProsRISK** | | | |
|  | **SN** | **SP** | **PPV** | **NPV** | **SN** | **SP** | **PPV** | **NPV** |
| **0.010** | 0.85 | 0.783 (0.664-0.869) | 0.038 | 0.998 | 0.85 | 0.900 (0.799-0.953) | 0.079 | 0.998 |
| **0.015** | 0.85 | 0.783 (0.664-0.869) | 0.056 | 0.997 | 0.85 | 0.900 (0.799-0.953) | 0.115 | 0.997 |
| **0.020** | 0.85 | 0.783 (0.664-0.869) | 0.074 | 0.996 | 0.85 | 0.900 (0.799-0.953) | 0.148 | 0.996 |
| **0.025** | 0.85 | 0.783 (0.664-0.869) | 0.091 | 0.995 | 0.85 | 0.900 (0.799-0.953) | 0.179 | 0.995 |
| **0.030** | 0.85 | 0.783 (0.664-0.869) | 0.108 | 0.994 | 0.85 | 0.900 (0.799-0.953) | 0.208 | 0.994 |

Abbreviations: PPV, positive predictive value; NPV, negative predictive value; SN, sensitivity; SP, specificity.

**TABLE S9** Positive and negative predictive values of the ProsRISK for Benign vs PCA in the validation set.

| **Prevalence** | **Benign vs PCA I-II** | | | | **Benign vs PCA Ⅲ-Ⅳ** | | | |
| --- | --- | --- | --- | --- | --- | --- | --- | --- |
|  | **ProsRISK** | | | | **ProsRISK** | | | |
|  | **SN** | **SP** | **PPV** | **NPV** | **SN** | **SP** | **PPV** | **NPV** |
| **0.010** | 0.698 (0.549-0.814) | 0.80 | 0.034 | 0.996 | 0.704 (0.515-0.842) | 0.80 | 0.034 | 0.996 |
| **0.015** | 0.698 (0.549-0.814) | 0.80 | 0.051 | 0.994 | 0.704 (0.515-0.842) | 0.80 | 0.051 | 0.994 |
| **0.020** | 0.698 (0.549-0.814) | 0.80 | 0.067 | 0.992 | 0.704 (0.515-0.842) | 0.80 | 0.067 | 0.993 |
| **0.025** | 0.698 (0.549-0.814) | 0.80 | 0.082 | 0.990 | 0.704 (0.515-0.842) | 0.80 | 0.083 | 0.991 |
| **0.030** | 0.698 (0.549-0.814) | 0.80 | 0.097 | 0.988 | 0.704 (0.515-0.842) | 0.80 | 0.098 | 0.989 |

Abbreviations: PPV, positive predictive value; NPV, negative predictive value; SN, sensitivity; SP, specificity.
